# Cell-type-informed genotyping of mosaic focal epilepsies reveals cell-autonomous and non-cell-autonomous disease-associated transcriptional programs

**DOI:** 10.1073/pnas.2509622122

**Published:** 2025-07-17

**Authors:** Sara Bizzotto, Maya Talukdar, Edward A. Stronge, Rosita B. Ramirez, Yingxi Yang, August Yue Huang, Qiwen Hu, Yingping Hou, Norma K. Hylton, Benjamin Finander, Ashton Tillett, Zinan Zhou, Brian H. Chhouk, Alissa M. D’Gama, Edward Yang, Timothy E. Green, David C. Reutens, Saul A. Mullen, Ingrid E. Scheffer, Michael S. Hildebrand, Russell J. Buono, Ingmar Blümcke, Annapurna H. Poduri, Sattar Khoshkhoo, Christopher A. Walsh

**Affiliations:** ^a^Division of Genetics and Genomics, Manton Center for Orphan Disease Research, Boston, MA 02115; ^b^Department of Pediatrics, Boston Children’s Hospital, Harvard Medical School, Boston, MA 02115; ^c^Broad Institute of Massachusetts Institute of Technology and Harvard, Cambridge, MA 02142; ^d^Imagine Institute, Team Somatic Mosaicism in Neurodevelopment and Disease, Université Paris Cité, Paris 75015, France; ^e^Harvard/Massachusetts Institute of Technology MD-PhD Program, Harvard Medical School, Boston, MA 02115; ^f^Department of Neurology, Brigham and Women’s Hospital, Harvard Medical School, Boston, MA 02115; ^g^Department of Biomedical Informatics, Harvard Medical School, Boston, MA 02115; ^h^Epilepsy Genetics Program, Division of Epilepsy and Neurophysiology, Department of Neurology, Boston Children’s Hospital, Harvard Medical School, Boston, MA 02115; ^i^Division of Newborn Medicine, Department of Pediatrics, Boston Children’s Hospital, Harvard Medical School, Boston, MA 02115; ^j^Department of Radiology, Boston Children’s Hospital, Boston, MA 02115; ^k^Department of Medicine (Austin Health), University of Melbourne, Heidelberg, VIC 3084, Australia; ^l^Centre for Advanced Imaging, The University of Queensland and Royal Brisbane and Women’s Hospital, Herston, QLD 4067, Australia; ^m^Neuroscience Group, Murdoch Children’s Research Institute, Parkville, VIC 3052, Australia; ^n^Department of Biomedical Sciences, Cooper Medical School of Rowan University, Camden, NJ 08103; ^o^Department of Neuropathology, University Hospitals Erlangen, Erlangen 91054, Germany; ^p^Epilepsy Center, Cleveland Clinic, Cleveland, OH 44106; ^q^Allen Discovery Center for Human Brain Evolution, Boston Children’s Hospital, Harvard Medical School, Boston, MA 02115; ^r^HHMI, Boston Children's Hospital, Boston, MA 02115

**Keywords:** epilepsy, cerebral cortex, MTOR, somatic mutation, development

## Abstract

While it is widely accepted that somatic variants that activate the PI3K-mTOR pathway are a major cause of drug-resistant focal epilepsy, typically associated with focal cortical dysplasia (FCD) type 2, understanding the mechanism of epileptogenesis requires identifying genotype-associated changes at the single-cell level, which is technically challenging with existing methods. Here, we performed single-nucleus RNA-sequencing (snRNA-seq) of 18 FCD type 2 samples removed surgically for treatment of drug-resistant epilepsy, and 17 non-FCD control samples, and analyzed additional published data comprising >400,000 single nuclei. We also performed simultaneous single-nucleus genotyping and gene expression analysis using two independent approaches: 1) a method that we called genotyping of transcriptomes enhanced with nanopore sequencing (GO-TEN) that combines targeted cDNA long-read sequencing with snRNA-seq, 2) ResolveOME snRNA-seq and DNA genotyping. snRNA-seq showed similar cell identities and proportions between cases and controls, suggesting that mosaic pathogenic variants in PI3K-mTOR pathway genes in FCD exert their effect by disrupting transcription in conserved cell types. GO-TEN and ResolveOME analyses confirmed that pathogenic variant-carrying cells have well-differentiated neuronal or glial identities, with enrichment of variants in cells of the neuroectodermal lineage, pointing to cortical neural progenitors as possible loci of somatic mutation. Within FCD type 2 lesions, we identified upregulation of PI3K-mTOR signaling and related pathways in variant-carrying neurons, downregulation of these pathways in non-variant-carrying neurons, as well as associated changes in microglial activation, cellular metabolism, synaptic homeostasis, and neuronal connectivity, all potentially contributing to epileptogenesis. These genotype-specific changes in mosaic lesions highlight potential disease mechanisms and therapeutic targets.

## Significance

Focal cortical dysplasia (FCD) type 2 is a malformation of the cerebral cortex causing drug-resistant epilepsy that can be due to somatic variants in PI3K-mTOR pathway genes giving rise to a mosaic lesion. It remains unclear when pathogenic variants causing FCD type 2 occur during development, and how they affect mutant compared to nonmutant cells. In this study, single-cell transcriptomics combined with cell-type-resolved genotyping of somatic variants reveal that FCD type 2 affects mainly neurectoderm-derived cortical cells. Mutant neurons in the mosaic cortical lesions show upregulation of pathways related to metabolism and cell growth, which are downregulated in nonmutant neurons. We further identified changes in microglial activation, synaptic homeostasis, and neuronal connectivity potentially contributing to epileptogenesis.

Focal cortical dysplasia (FCD) spectrum disorders represent cortical malformations that occur in the absence of other organ system involvement and are the most common cause of drug-resistant epilepsy requiring neurosurgical treatment in children (1). FCDs are developmental brain lesions that result from pathogenic somatic variants, and are unilateral or more commonly restricted to a small cortical region (2, 3). The most common FCD subtypes—FCD type 2A (FCD2A), FCD type 2B (FCD2B), and hemimegalencephaly (HME)—are characterized by localized regions of abnormal cortex appearing at the histopathological level as dyslamination of the six-layer structure and the presence of rare, maloriented, dysmorphic neurons (DNs). In FCD2B and HME, another hallmark finding are balloon cells (BCs), which are rare, enlarged, oval-shaped, eosinophilic cells with one or more nuclei (4). A related condition, tuberous sclerosis complex (TSC), is characterized by multiple dysplastic cortical lesions with similar histopathology, commonly referred to as cortical tubers (5).

Pathogenic somatic variants in FCD2 often result in hyperactivation of phosphatidylinositol-3 Kinase (PI3K)-mechanistic target of rapamycin (mTOR) pathway, which regulates cell growth, metabolism, proliferation, and survival (6). FCD2-associated lesions often harbor gain-of-function (GoF) somatic heterozygous variants in the activators of this pathway, such as *AKT3*, *PIK3CA*, *RHEB,* and *MTOR* (3, 7). Additionally, somatic loss-of-function (LoF) variants have been reported in PI3K-mTOR pathway repressor genes such as *TSC1/2*, *DEPDC5*, *NPRL2/3,* and *PTEN.* Somatic LoF variants are usually found in patients with a germline LoF variant in the second allele, thus causing mosaic biallelic LoF reminiscent of Knudson’s second hit model for tumor suppressors (2, 3, 8). FCD2 and HME are genetically and histopathologically very similar lesions, with the hemispheric extent and the larger fraction of variant-carrying cells in HME potentially indicating an earlier developmental stage at which the variants occur.

While up to 80% of dysplasias with FCD2 pathology show activating PI3K-mTOR variants (9), how the genetic variants affect cell identities and states in the brain, and how these effects might lead to drug-resistant epilepsy, is unclear. Furthermore, it is unknown when and how during development these variants occur. To address these key unanswered questions, we performed single-nucleus RNA sequencing (snRNA-seq) on surgical specimens obtained from a cohort of 17 patients clinically diagnosed with FCD2 spectrum pathology, 15 of which carried a variety of previously reported PI3K-mTOR pathway-associated variants (3, 9). We show that FCD2 lesions do not contain any novel cell types other than those found in control brains at detectable frequency, nor do they display consistent shifts in cell type composition. Second, we performed cell-type-resolved single-nucleus genotyping and genotype-informed transcriptional analysis of FCD2 tissue by applying two orthogonal methods: 1) well-based ResolveOME (10) parallel RNA sequencing and DNA analysis, and 2) droplet-based genotyping of transcriptome enhanced with nanopore sequencing (GO-TEN), a method we developed that allows genotyping of targeted loci in single nuclei cDNA, while also obtaining their full transcriptional profiles. Both methods showed that pathogenic variant-carrying cells map to well-differentiated cell types of the cortical neural lineage, consistent with variants occurring after neuroectodermal specification. Finally, transcriptomic comparison of variant-carrying and non-variant-carrying nuclei revealed alterations to cell-autonomous as well as global non-cell-autonomous gene expression and cell–cell connectivity, potentially contributing to epileptogenesis and FCD2 pathophysiology.

## Results

### Cell Type Analysis of FCD2 Brains.

**Fig. 1.**
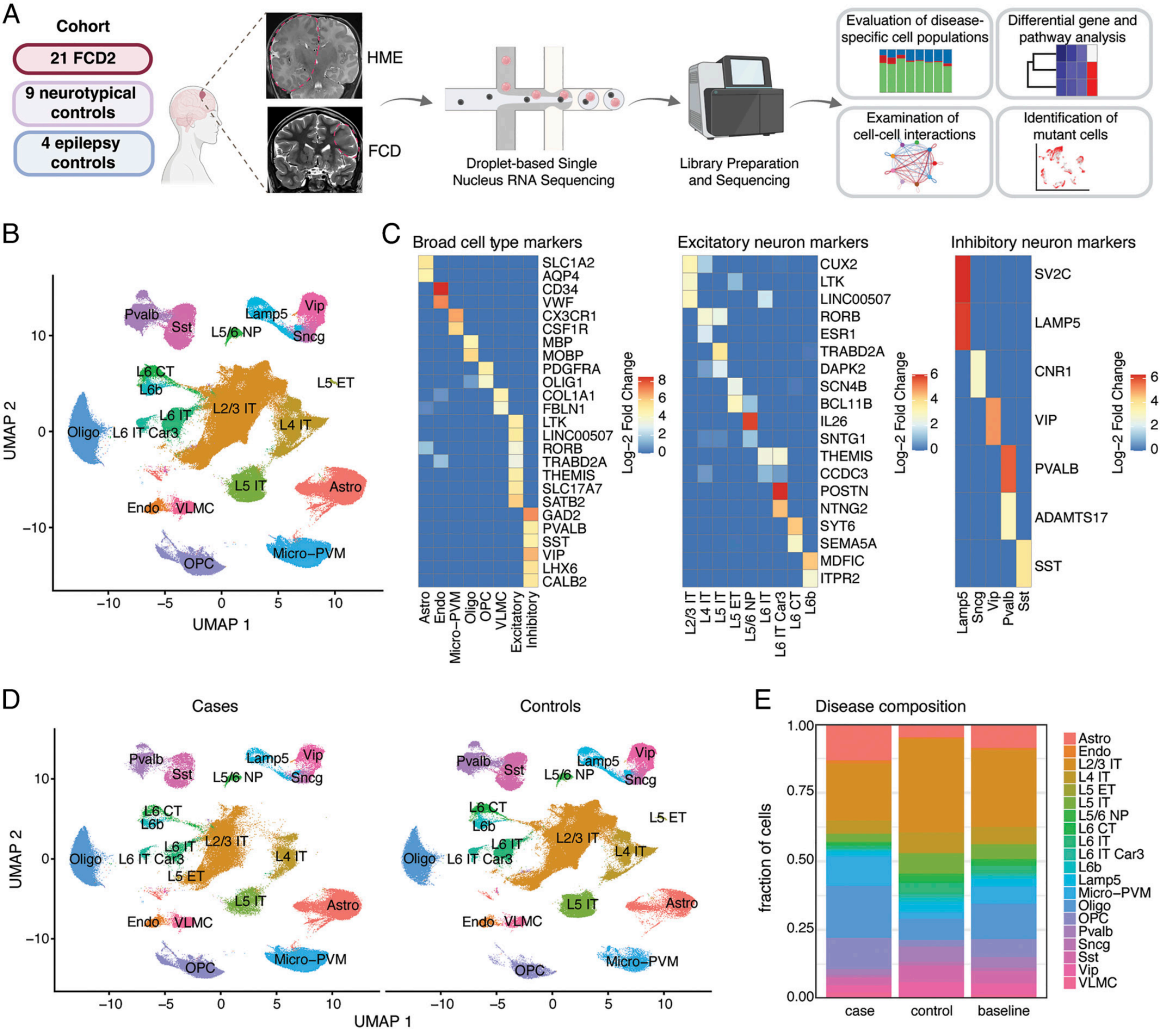
Conserved cortical cell types between control and FCD2 brains. (*A*) Schematic of the experimental and analytical workflow. Representative coronal T2 MRI images for HME (E174) and FCD2B (E364) demarcate the abnormality with dashed red lines. (*B*) Uniform manifold approximation and projection (UMAP) showing cell type clusters identified in the integrated [Harmony (16)] case and control snRNA-seq atlas. (*C*) Expression of canonical brain cell type marker genes. (*D*) Conserved cell type clusters between cases and controls. (*E*) Cell type composition of case and control groups compared to the baseline (all case and control nuclei). Chi-squared proportion test (*P* = 0.2358).

Histopathological hallmarks of FCD2 include the presence of DNs expressing NeuN and GFAP, perikaryal expression of neurofilaments (SMI31), excess phosphorylated ribosomal protein S6 (pS6), and in certain subtypes eosinophilic BCs (4). In order to determine the cell types and identities present in FCD2 lesions and whether DNs and BCs are transcriptionally distinguishable, we performed snRNA-seq (11) on 18 fresh-frozen surgically resected brain samples from 17 individuals with FCD2 spectrum lesions, including ten FCD2 and seven HME (two samples were obtained from the same individual), and compared them with control snRNA-seq data from nonlesional brains (Fig. 1*A*, *Methods*, *SI Appendix*, Table S1 and Fig. S1 *A* and *B*, and Dataset S1). Diagnosis of FCD2 for all the samples analyzed was made in multiple expert epilepsy surgical centers (Boston Children’s Hospital, Cleveland Clinic, Austin Health, and European Epilepsy Brain Bank) based on magnetic resonance imaging (MRI) and histopathological assessment of the tissue removed during surgery, confirming the presence of dysplastic cells (examples are reported in *SI Appendix*, Fig. S1*A*). The non-FCD controls consisted of 1) anterior temporal cortex (Brodmann area BA38) surgical resections from four patients with mesial temporal lobe epilepsy (mTLE) as nonlesional epilepsy controls, 2) two prefrontal cortex (BA9) samples previously published by our lab (12), and 3) nine occipital cortex samples (primary and secondary visual cortices, BA17 and BA18) (13) and two temporal neocortex samples from a total of four neurotypical postmortem brains. Additionally, we analyzed snRNA-seq data obtained from the BRAIN Initiative Cell Census Network Human Brain Cell Atlas v1.0 (14) corresponding to seven areas matching those sampled in our data (*SI Appendix*, Table S1) and obtained from four different individuals. Finally, we analyzed cortical tissue previously published by Chung et al. (15) consisting of one neurotypical control and four FCD2 cases. Thus, we analyzed in total 22 FCD2 samples from 21 individuals, four epilepsy control samples from four different individuals, and 21 neurotypical samples from nine individuals (*SI Appendix*, Table S1 and Fig. 1*A*), for a total of 406,699 single nuclei that passed quality control filters: 186,166 nuclei from cases and 220,533 nuclei from controls (*SI Appendix*, Fig. S1*C* and *Methods*).

Pathogenic variants were previously identified (3, 9) in 15 FCD2 samples in our cohort, including one *TSC1*, four *PIK3CA,* and eight *MTOR* somatic variants, one somatic copy number gain in the long (q) arm of chromosome 1 including *AKT3*, and one *DEPDC5* germline variant. Somatic variants showed bulk variant allele frequencies (VAFs) spanning 2.3 to 25.8% (*SI Appendix*, Table S1). Since all the *MTOR* (17) and *PIK3CA* (18) variants in our cohort have been previously shown through functional studies to increase PI3K-mTOR activity, we combined the FCD2 cases for downstream analyses to maximize statistical power.

All case and control samples were processed and integrated after filtering out putative doublets (19) and low-quality nuclei, and controlling for ambient RNA contamination (20). Cell-type annotation was then performed using a semisupervised approach consisting in a first label-transfer analysis using the reference Allen Institute motor cortex snRNA-seq atlas (21), and a subsequent annotation refinement by examining expression patterns of canonical brain cell type marker genes (14, 22) (Fig. 1 *B* and *C*, *Methods*, *SI Appendix*, and *SI Appendix*, Fig. S2). Data from different sources contained all cell clusters, indicative of efficient integration (*SI Appendix*, Fig. S3*A*). We identified all major cortical cell types including nine excitatory neuron subtypes, five inhibitory neuron subtypes, oligodendrocyte precursor cells (OPCs), mature oligodendrocytes, astrocytes and microglia-perivascular macrophages (PVM), vascular and leptomeningeal cells (VLMC), and endothelial cells. Excitatory neuron subtypes included layer (L) 2/3 intratelencephalic (IT), L4 IT, L5 IT, L6 IT, L6 IT Car3, L5 extratelencephalic (ET), L5/6 near-projecting (NP), L6 cortico-thalamic (CT), and L6b neurons. Inhibitory neuron subtypes included Sst, Lamp5, Sncg, Pvalb, and Vip (Fig. 1 *B* and *C*). All clusters in this integrated snRNA-seq atlas contained nuclei from both cases and controls, and we could not identify distinguishable new clusters specific to FCD2 cases representing nuclei of an abnormal or novel identity like DNs and/or BCs (Fig. 1*D*). It is important to note that DNs and BCs, while cytologically impressive, represent rare cell types in histological sections (*SI Appendix*, Fig. S1*A*).

No statistically significant difference was found in global cell type composition in FCD2 brains compared to controls (chi-squared proportion test; *P* = 0.2358; Fig. 1*E* and *Methods*). Reasoning that such global composition analyses may obscure more subtle shifts in proportions of a small number of cell types, we compared case and control group contributions to each cell type using propeller (23) and found a significant enrichment in the case group of microglia-PVM (adj. *P* = 0.0003), OPCs (adj. *P* = 0.0006), endothelial cells (adj. *P* = 0.0024), and astrocytes (adj. *P* = 0.0128, *SI Appendix*, Fig. S3*B*, Dataset S2, and *Methods*). This may be suggestive of microglial activation and gliosis (24) and/or reflect lower neuronal density (25) in FCD2 tissue. However, our analysis does not account for potential sampling biases that may impact these results.

The absence of clusters containing dysplastic cells may be due to reasons such as integration, rarity of dysplastic cells or doublet removal. We evaluated these possibilities by 1) integration of FCD2 samples using different methods (16, 26) (*SI Appendix* and *SI Appendix*, Figs. S4–S6); 2) analysis restricted to four cases carrying a pathogenic variant with high VAF (*SI Appendix*, Fig. S7 and *SI Appendix*); 3) data analysis without doublet filtering (*SI Appendix*, Fig. S8 and *SI Appendix*). Together these analyses (described in detail in *SI Appendix*) suggest that the morphological alterations that characterize DNs and BCs in FCD2 tissue do not necessarily represent new cell identities as determined by gene expression. Dysplastic cells may also be lost during nuclei preparation due to fragility or other unique physical properties, although this hypothesis cannot be directly tested.

### Altered Energy Metabolism and Microglial Immune Activation in FCD2.

**Fig. 2.**
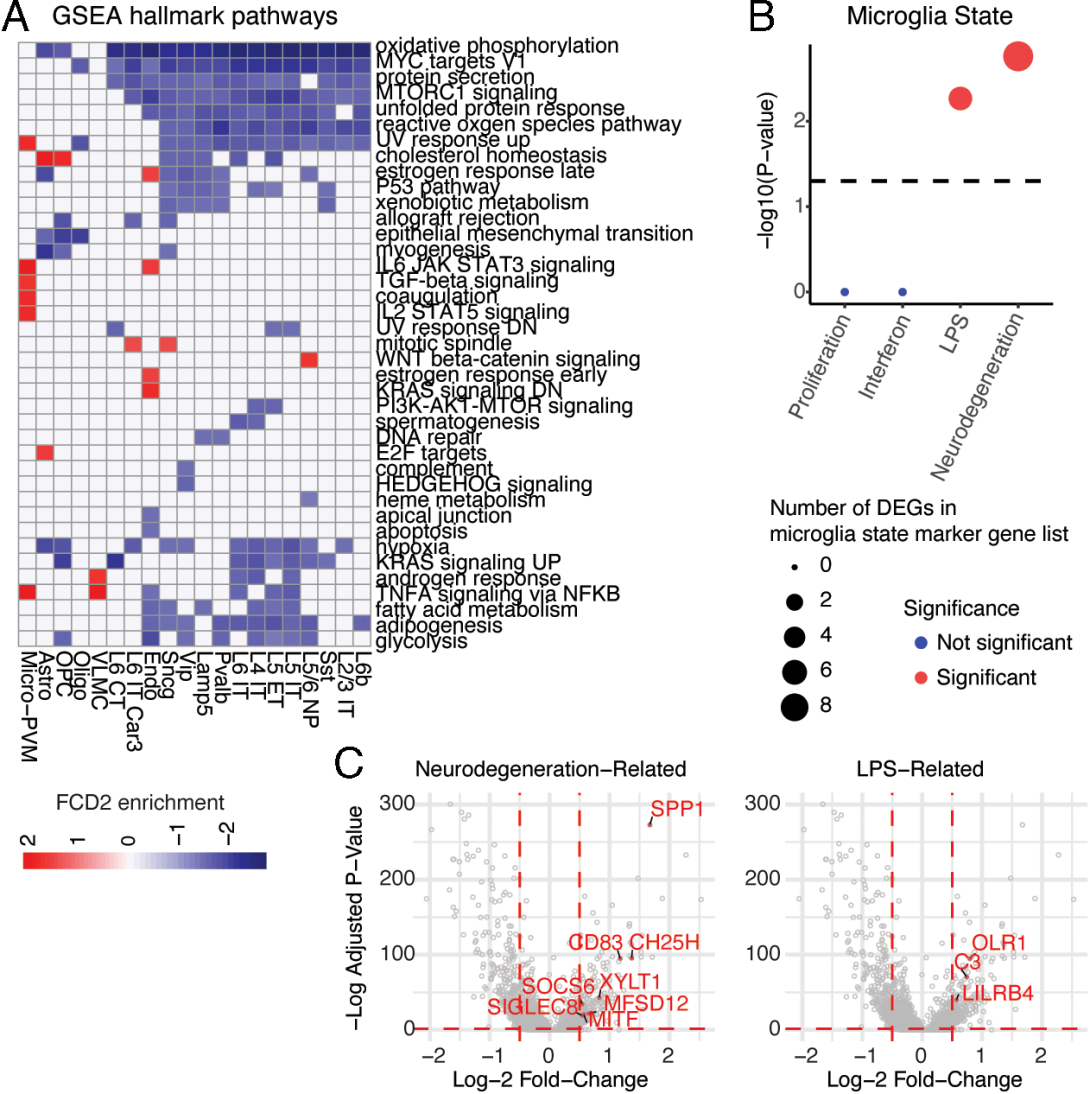
Alteration of energy metabolism and microglial immune activation in FCD2. (*A*) Heatmap of Molecular Signatures Database Hallmark pathways (33, 34) enriched in cases compared to controls per cell type, obtained with fgsea (35) (Dataset S3). (*B*) Enrichment of DEGs in case vs. control microglia for various microglial state marker genes (36). *P*-values, hypergeometric test with a Benjamini–Hochberg correction for multiple hypothesis testing. Significance denoted for adj. *P* < 0.05. (*C*) Volcano plots of DEGs driving enrichment of neurodegeneration and lipopolysaccharide-activation-associated states depicted in (*B*). Wilcoxon rank sum test with a Benjamini–Hochberg correction for multiple hypothesis testing. DEGs with an average log-2 fold-change > 0.5 and adj. *P* < 0.05 are highlighted.

Differential gene expression (DGE) and gene set enrichment analyses (GSEA) characterized cell-type-associated changes in transcriptional programs between cases and controls (*SI Appendix*, Fig. S9, Datasets S3 and S4; *Methods*). We found down-regulation of pathways associated with energy metabolism, cellular growth and proliferation, and biosynthesis, such as oxidative phosphorylation, MYC targets V1, PI3K-AKT-MTOR signaling, MTORC1 signaling, reactive oxygen species pathway, hypoxia, KRAS signaling, fatty acid metabolism, adipogenesis, and glycolysis, across most neuronal subtypes (Fig. 2*A*, *SI Appendix*, Fig. S10*A* and Datasets S3 and S4). Alterations in energy metabolism and biosynthesis may significantly alter key neuronal properties such as axonal transport and synaptic function (27). The seemingly paradoxical downregulation of these pathways is consistent with the frequent hypometabolism observed in FCD lesions by functional MRI (28–30) and may reflect a compensatory reaction to overactivation of this pathway in variant-carrying cells (see below). Pseudobulk DGE and GSEA for all nuclei, neuronal nuclei only, and glial nuclei only revealed that KRAS and MYC pathways were downregulated in all nuclei, while most other pathways related to PI3K-mTOR signaling such as MTORC1 signaling were not differentially regulated overall, even though specifically and significantly downregulated in neurons (*SI Appendix*, Fig. S10*B* and Dataset S5). Our pseudobulk analysis provides a possible explanation for why prior bulk RNA-seq studies (31, 32) did not observe some of the cell-type-specific transcriptional changes reported here, and highlights a potential benefit of utilizing snRNA-seq to study cellularly heterogeneous human FCD2 tissue.

GSEA also revealed FCD2-associated immune activation in microglia, where we found upregulation of terms such as IL2-STAT5 signaling, TGF-beta signaling, TNFA signaling via NFKB and IL6-JAK-STAT3 signaling (Fig. 2*A* and *SI Appendix*, Fig. S10*A*). Furthermore, we identified a significant overlap between differentially expressed genes (DEGs) that were upregulated in FCD2 microglia, and microglial states associated with LPS-induced immune activation and neurodegeneration (36) (Fig. 2*B*). Among the most significantly upregulated neurodegeneration-related genes we found *SPP1*, *CD83*, *CH25H*, *XYLT1*, *MFSD12*, *MITF*, *SOCS6,* and *SIGLEC8*, while for LPS-induced immune activation we found *OLR1*, *C3,* and *LILRB4* (Fig. 2*C*). Taken together, our data highlight a role for microglial activation in FCD2 pathology that may be a cause or a consequence of epileptic seizures, and that mirrors microglial activation seen in other neurological disorders (37).

Finally, to assess whether the observed transcriptional changes were specific to PI3K-mTOR overactivation vs. changes generally caused by chronic epilepsy, we compared FCD2 cases specifically to nonlesional temporal neocortical resection samples from individuals with mTLE, and thus exposed to recurrent seizures, without any overt genetic or histopathologic abnormalities. Consistent with our prior analyses, we found evidence of microglial activation such as upregulation of TNFA signaling in FCD2 cases vs mTLE controls, suggesting that this may be an FCD2-specific effect (Datasets S6 and S7 and *SI Appendix*, Fig. S10*C*). This is in line with a mosaic mouse model of epilepsy showing that FCD-associated microglial density is higher in regions containing mutant cells compared to the contralateral hemisphere (38). We also detected significant differences in key pathways involved in transcriptional regulation such as MYC and E2F signaling in several neuronal subtypes and astrocytes (*SI Appendix*, Fig. S10*C*). The direction of these changes, however, differed when comparing against neurotypical vs mTLE controls, implying that these pathways have broader roles in seizures beyond their direct interaction with PI3K-mTOR signaling (39, 40).

### Changes in Neuronal Signaling in FCD2.

**Fig. 3.**
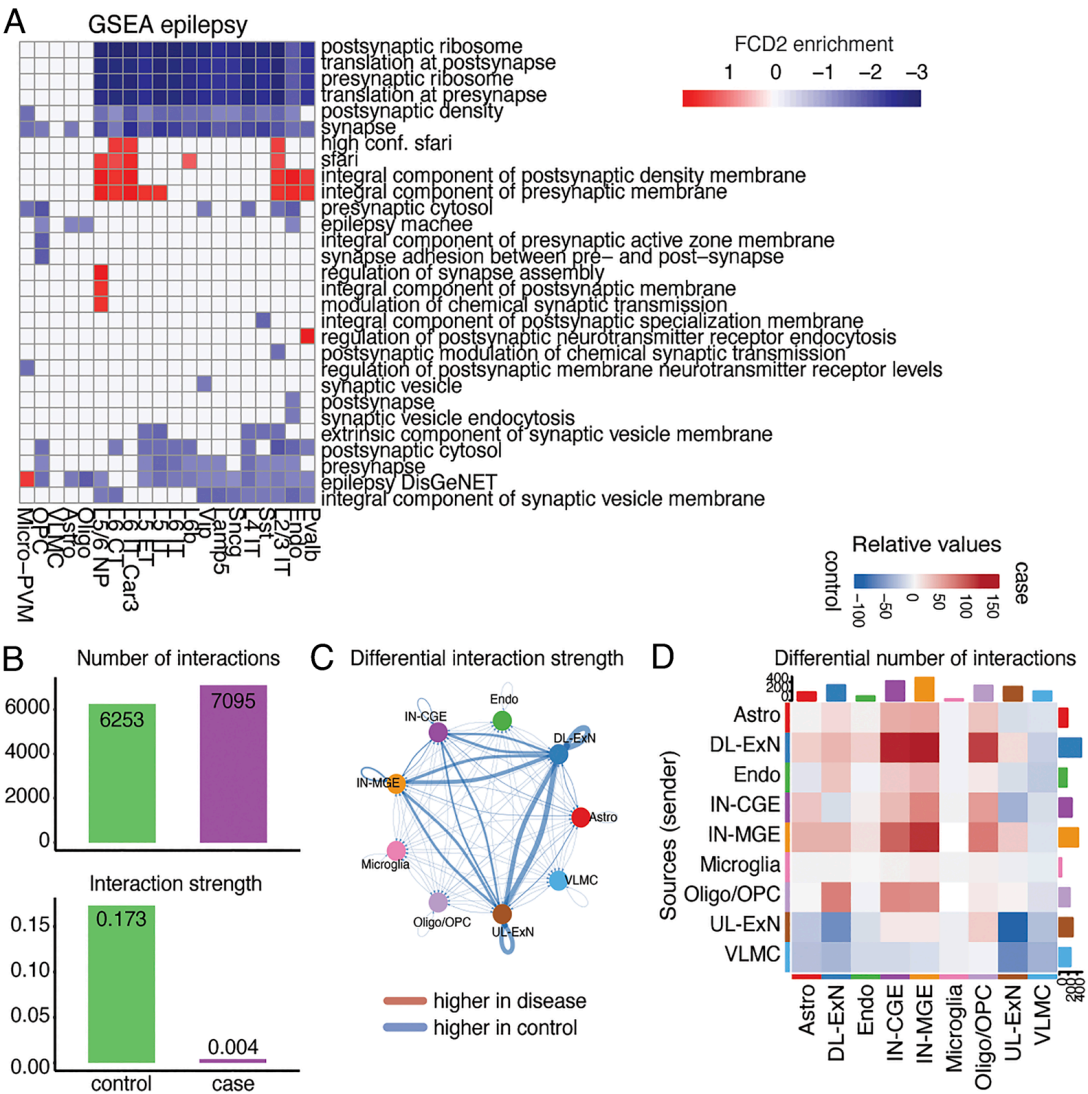
Altered signaling between neuronal subtypes in FCD2. (*A*) Heatmap showing pathways enriched in cases compared to controls per cell type, obtained with fgsea (35) and epilepsy-related ontologies (41–44). (*B*) Bar plots displaying overall number of interactions and interaction strength predicted by CellChat (46) in cases vs. controls. (*C*) Circos plot where edge thickness represents the case vs. control differential interaction strength predicted by CellChat. (*D*) Heatmap displaying differential number of interactions per cell type in cases vs. controls obtained with CellChat. Sender cell types are reported on the y-axis.

In order to identify mechanisms potentially contributing to epileptic seizures, we performed GSEA by interrogating specific gene ontologies such as the synaptic gene ontology (SynGO) database (41), as well as gene ontologies associated with epilepsy based on Macnee et al. (42), DisGeNET (https://www.disgenet.com) (43), and the Simons Foundation Autism Research Initiative (SFARI) database (https://gene.sfari.org) (44) (*Methods*), epilepsy being a known comorbidity for autism spectrum disorder (ASD) (45). In neurons in the disease group, we found downregulation of terms related to synaptic translation. SFARI genes were upregulated in several excitatory neuron subtypes such as L5/6 NP, L6 CT, L6 IT Car3, and L2/3 IT. Upregulation of terms related to synapse assembly and modulation of synaptic transmission was also found in neurons. Finally, epilepsy DisGeNET was specifically upregulated in microglia, again suggesting an involvement in FCD2 pathology (Fig. 3*A* and *SI Appendix*, Fig. S11*A*). We found very little differences in pathway enrichment between FCD2 and mTLE, which is expected, since this analysis is biased toward epilepsy-related pathways (*SI Appendix*, Fig. S11*B*).

Analysis with CellChat, a tool that quantitatively infers intercellular communication networks from scRNA-seq data based on the expression of known receptor/ligand pairs (46) (*Methods* and *SI Appendix*), revealed changes in intercellular signaling that could explain FCD2-related seizure susceptibility. ChellChat analysis performed on nine major cellular categories—1) deeper-layer excitatory neurons (DL-ExN, including L4 IT, L5 IT, L5 ET, L5/6 NP, L6 CT, L6 IT, L6 IT Car3, and L6b), 2) upper-layer excitatory neurons (UL-ExN, including only L2/3 IT), 3) caudal ganglionic eminence (CGE)-derived interneurons (IN-CGE, made of Vip, Sncg, and Lamp5), 4) medial ganglionic eminence (MGE)-derived interneurons (IN-MGE, including Sst and Pvalb) (47), 5) endothelial cells, 6) astrocytes, 7) VLMC, 8) oligodendrocytes/OPC, and 9) microglia—revealed a very slight increase in the number of inferred interactions (interpretable as the number of connections) in cases compared to controls, but with a marked decrease in overall interaction strengths (interpretable as connection stability, Fig. 3*B*). In particular, in cases the interaction strength was decreased in both excitatory–excitatory neurons and excitatory-inhibitory neuron connections (Fig. 3*C*), while at the same time, the number of interactions between DL-ExN and interneurons increased in cases (Fig. 3*D* and *SI Appendix*, Fig. S12*A*). These changes in connectivity between neuronal subtypes in FCD2 may contribute to the circuit disruption and hyperexcitability that are observed in epilepsy. Some of the major signaling pathways that might account for differential connectivity in neurons between cases and controls include those involved in synaptic development and signaling, such as neurexins (NRXN), adhesion G protein–coupled receptor L (ADGRL), protein tyrosine phosphatases receptor (PTPR), neuronal growth regulator (NEGR), neuregulin (SRG), and glutamate (*SI Appendix*, Fig. S12 *B* and *C* and Dataset S8).

### Cell Type–Informed Genotyping of Pathogenic Somatic variants.

**Fig. 4.**
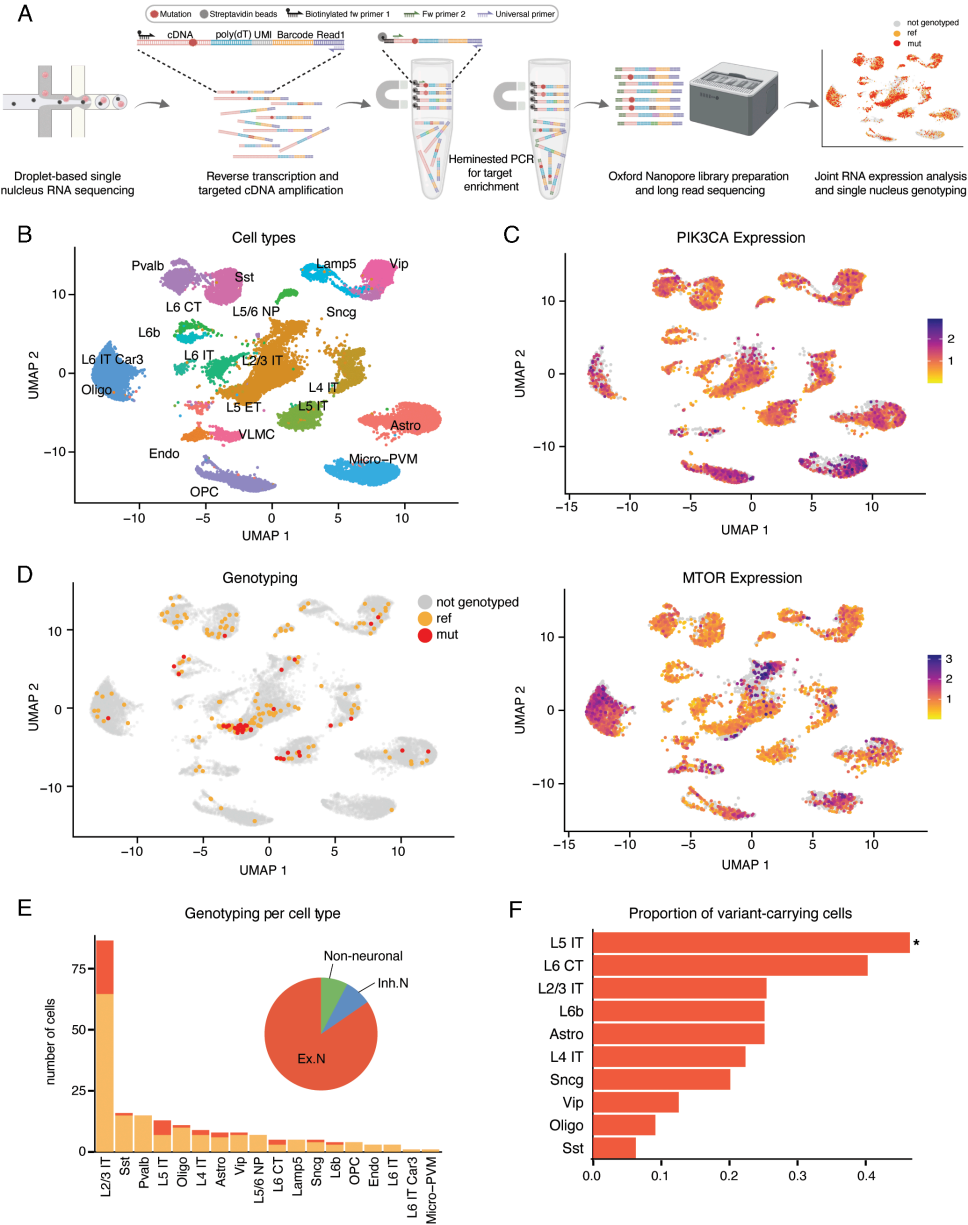
GO-TEN cell type–informed genotyping of pathogenic somatic variants in FCD2. (*A*) Schematic of the GO-TEN experimental workflow. (*B*) UMAP representation of cell types in integrated snRNA-seq atlas. (*C*) UMAP representation of *PIK3CA* and *MTOR* gene expression in integrated snRNA-seq atlas. (*D*) UMAP representation of *PIK3CA* and *MTOR* GO-TEN nuclei genotyped by the high-specificity approach. Data from three cases (one *PIK3CA* and two *MTOR*) were pooled together. (*E*) Number of nuclei genotyped with GO-TEN high-specificity approach as ref or mut. The pie chart shows the contribution of broad cell categories to variant-carrying nuclei. (*F*) Number of mut nuclei, normalized for the total number of genotyped nuclei for each variant-carrying cell type. Cell types enriched for mut nuclei are indicated with a * (hypergeometric test, *P* < 0.05; Dataset S14).

Identifying cell states associated with the pathogenic genotype in mosaic neurological conditions represents a technological hurdle as it requires sequencing both DNA and RNA from single cells (48), although newly developed technologies (49–51) have shown some success. Here, we developed GO-TEN, which modifies genotyping of transcriptome (GoT) (49) to combine 10X snRNA-seq with an orthogonal genotyping strategy based on Oxford Nanopore Technology (ONT) long-read sequencing, allowing the genotyping of variant loci located multiple kilobases away from the 3′-UTRs within a transcript. Similar to GoT, GO-TEN can be applied to the full-length cDNA that did not undergo fragmentation and library preparation, obtained from an intermediate step in the 10X Genomics snRNA-seq protocol, and entails generation of on-target amplicons that capture the cell barcodes (CBCs) and unique molecular identifiers (UMIs) for the transcripts, as well as the genomic locus corresponding to the variant of interest. The CBCs are used to map genotyped cells to the Illumina short-read snRNA-seq data obtained from the same cDNA pool (Fig. 4*A* and *Methods*). While GO-TEN has technical similarities to GOT-Splice (52), it was specifically optimized to study variants in the long and relatively lowly expressed *PIK3CA* and *MTOR* genes frequently implicated in FCD2 pathology. Moreover, compared to prior studies that attempted long-read sequencing of full-length cDNA directly (53, 54), GO-TEN has the benefit of a significantly higher number of on-target reads, making it possible to establish a statistical framework for consensus genotyping based on base quality as well as read and UMI-level support (*Methods* and *SI Appendix*) to achieve improved genotyping accuracy and efficiency.

GO-TEN generated single-cell genotyping data for three surgical samples in our cohort, carrying known recurrent activating missense PI3K-mTOR pathway variants with a range of VAFs. The first case (E174) was clinically diagnosed with HME with a *PIK3CA* p.E542K variant at ~30% mosaicism (bulk VAF = 13.9 to 17.4%) (3). The second case (FC5801) was diagnosed with FCD2B, and carried an *MTOR* p.C1483R variant with ~20% mosaicism (bulk VAF = 10.0 to 10.6%) (3). The third case (FC5501) was also diagnosed with FCD2B, and carried an *MTOR* p.L1460P variant with ~5% mosaicism (bulk VAF = 2.3 to 2.6%) (3). We performed genotyping using two methods: 1) a more stringent approach to maximize specificity by filtering ONT reads that do not contain an UMI found in the 10X Illumina snRNA-seq data for the genes of interest, and 2) a less stringent approach that does not require UMI match, and that maximizes sensitivity (*Methods* and *SI Appendix*). Known variant loci were genotyped using a custom adaptation of the Bayesian genotyper from MosaicHunter optimized for snRNA-seq data, scMosaicHunter (55, 56) (*Methods*, *SI Appendix*, Fig. S13 *A* and *B*, and *SI Appendix*).

The high-sensitivity approach genotyped 5,434 nuclei across all three cases (Datasets S9–S13) of which 3,636 showed only the reference allele (ref), while 1,798 carried the mutant allele (mut). 1,731 cells were genotyped for the *PIK3CA* locus, of which 1600 were ref, and 131 (7.6%) were mut. *MTOR* loci were genotyped in a total of 3,703 cells (626 for FC5801 and 3,077 for FC5501), of which 2,036 were ref (299 for FC5801 and 1,737 for FC5501), and 1,667 were mut (327 for FC5801 and 1,340 for FC5501). The fractions of mut cells in each *MTOR* case were 52.2% and 43.5% for FC5801 and FC5501, respectively. Analysis of covariates that may affect genotyping efficiency (*SI Appendix*) showed that gene expression level positively correlated with the probability of genotyping, as expected since GO-TEN genotypes cDNA. However, we also found some cell types with increased or decreased probability of being genotyped for specific gene variants (*SI Appendix*, Fig. S13*C*), potentially as a consequence of specific transcript isoforms that may be more or less efficiently captured by GO-TEN. Variant-carrying cell types, identified with extremely high confidence using the conservative high-specificity approach, included L2/3 IT, L5 IT, L4 IT, L6 CT, and L6b excitatory neuron subtypes, Sst, Vip, and Sncg interneuron subtypes, and glial cells such as oligodendrocyte and astrocyte (Fig. 4 *B*–*E* and Datasets S9–S13). This finding is consistent with the mutation arising in the neuroectodermal lineage and perhaps in a cortical neural progenitor cell. Although a few variant-carrying microglia and endothelial cells were identified with the high-sensitivity approach (*SI Appendix*, Fig. S14 and Datasets S9–S13), the low rate is consistent with false-positive calls. The shared presence of variants in both excitatory and inhibitory neurons could be interpreted as suggesting an origin of the mutation in an early forebrain founder progenitor, but more recent studies (13, 57–59) have suggested a shared origin of these neuronal types even relatively late in human cortical neurogenesis. Thus, we interpret variant-carrying cells as most likely consistent with the normal neural lineages, rather than being the product of selective bottlenecks (60) or lineages that are altered by the variants. Furthermore, GO-TEN shows that variant-carrying cells map with well-differentiated cell types, consistent with the absence of a dysplastic cell cluster in snRNA-seq data.

Normalizing the number of mut cells against the genotyped cells (ref plus mut) to control for differential genotyping among various cell types, showed significant enrichment of variants in L5 IT neurons compared to other variant-carrying cell types (hypergeometric test with Benjamini–Hochberg correction for multiple hypothesis testing, *P* = 0.0303, Dataset S14). The second cell type most enriched for variant-carrying cells was L6 CT, followed by L2/3 IT, L6b, astrocytes, L4 IT, Sncg, Vip, oligodendrocytes, and Sst (Fig. 4*F*). Although the two genotyping approaches give slightly different results in terms of cell types enriched for the pathogenic variants (Fig. 4*F* and *SI Appendix*, Fig. S14*D*)—perhaps attributable to the lower number of cells in the high-specificity approach and/or higher false positive rate from the high-sensitivity calls—the cell-type-informed genotyping overall suggests enrichment of pathogenic variants in excitatory neurons compared to other cortical cell types.

**Fig. 5.**
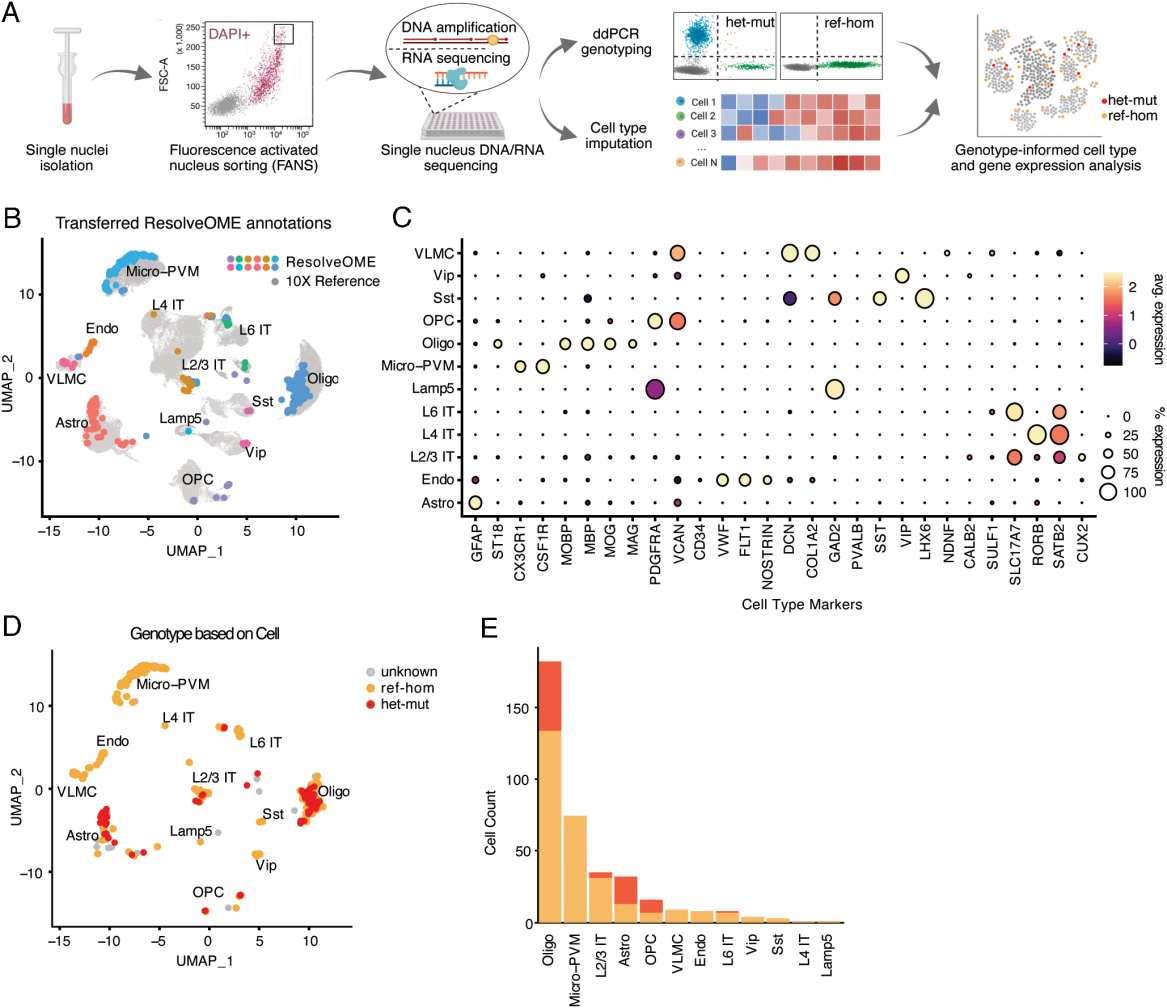
Combined single-nucleus DNA and RNA analysis in FCD2. (*A*) Schematic of the parallel DNA and RNA analysis using ResolveOME (10) combined with ddPCR. (*B*) UMAP displaying ResolveOME cell type clusters overlapping the integrated FCD2 reference snRNA-seq data. (*C*) Dot plot showing the expression of known cell type marker genes confirming label transfer from 10X snRNA-seq data. (*D*) UMAP showing *PIK3CA* and *MTOR* genotyped nuclei, colored as ref-hom and het-mut. Cells labeled as “unknown” did not meet ddPCR quality requirements (*Methods*). Data from five cases (four *PIK3CA* and one *MTOR*) were pooled together. (*E*) Barplot summarizing the number and cell type of confidently genotyped nuclei.

We performed parallel single-nucleus DNA and RNA analysis using ResolveOME (10), which combines whole-genome amplification (WGA) through primary template-directed amplification (PTA) with full-transcript reverse transcription, on DAPI sorted nuclei (Fig. 5*A*, *Methods*, and *SI Appendix*) from five cases in total, three of which were also included in our snRNA-seq cohort: two with *PIK3CA* variants (E174, E274) and one with an *MTOR* variant (E286; see *SI Appendix*, Table S1). Two additional cases had *PIK3CA* variants (EP39801 and EP41101; see *SI Appendix*, Table S1, Dataset S1, and *Methods*). We genotyped somatic variants in PTA-amplified single-cell DNA using droplet digital PCR (ddPCR) with probes specifically designed to identify the variants of interest (*SI Appendix*). This method gives very sensitive and high-fidelity genotyping of both alleles (Fig. 5*A*). In parallel, on the same nuclei used for genotyping, we performed whole-transcriptome sequencing. Out of 659 sequenced nuclei, we obtained data of sufficient RNA quality for 498 total nuclei, of which 372 nuclei were confidently genotyped: 50 from E174, 98 from E274, 126 from E286, 65 from EP39801, and 33 from EP41101. Of these, 81 total nuclei were heterozygous for the variant of interest (Datasets S15).

We used whole-transcriptome data obtained with ResolveOME to map each single-nucleus to the FCD2-only snRNA-seq dataset using label transfer (26), and subsequently validated the annotation by checking for expression of canonical cell type marker genes (Fig. 5 *B* and *C* and *SI Appendix*, Fig. S15). By combining this with ddPCR DNA genotyping, we could map reference homozygous (ref-hom) and heterozygous mutant (het-mut) nuclei across cell type clusters (Fig. 5*D*). As with GO-TEN, we found het-mut nuclei among L2/3 IT and L6 IT excitatory neurons, as well as OPCs, oligodendrocytes, and astrocytes (Fig. 5*E* and Dataset S15). We attribute the overrepresentation of glial cells in the ResolveOME experiment to oversampling of the white matter caused by variability in the available surgical tissue. We obtained genotyping information for a total of 74 microglia-PVM, 9 VLMC, and 8 endothelial cells but found no het-mut cells among them. Thus, our combined GO-TEN and ResolveOME data suggest that *PIK3CA* and *MTOR* somatic variants in FCD2, even when found at high VAF in the brain such as the one studied here, seem restricted to the neuroectodermal lineage, and are carried by cells that transcriptionally map with well-differentiated cell types.

### Genotype-Associated Transcriptional Alterations.

**Fig. 6.**
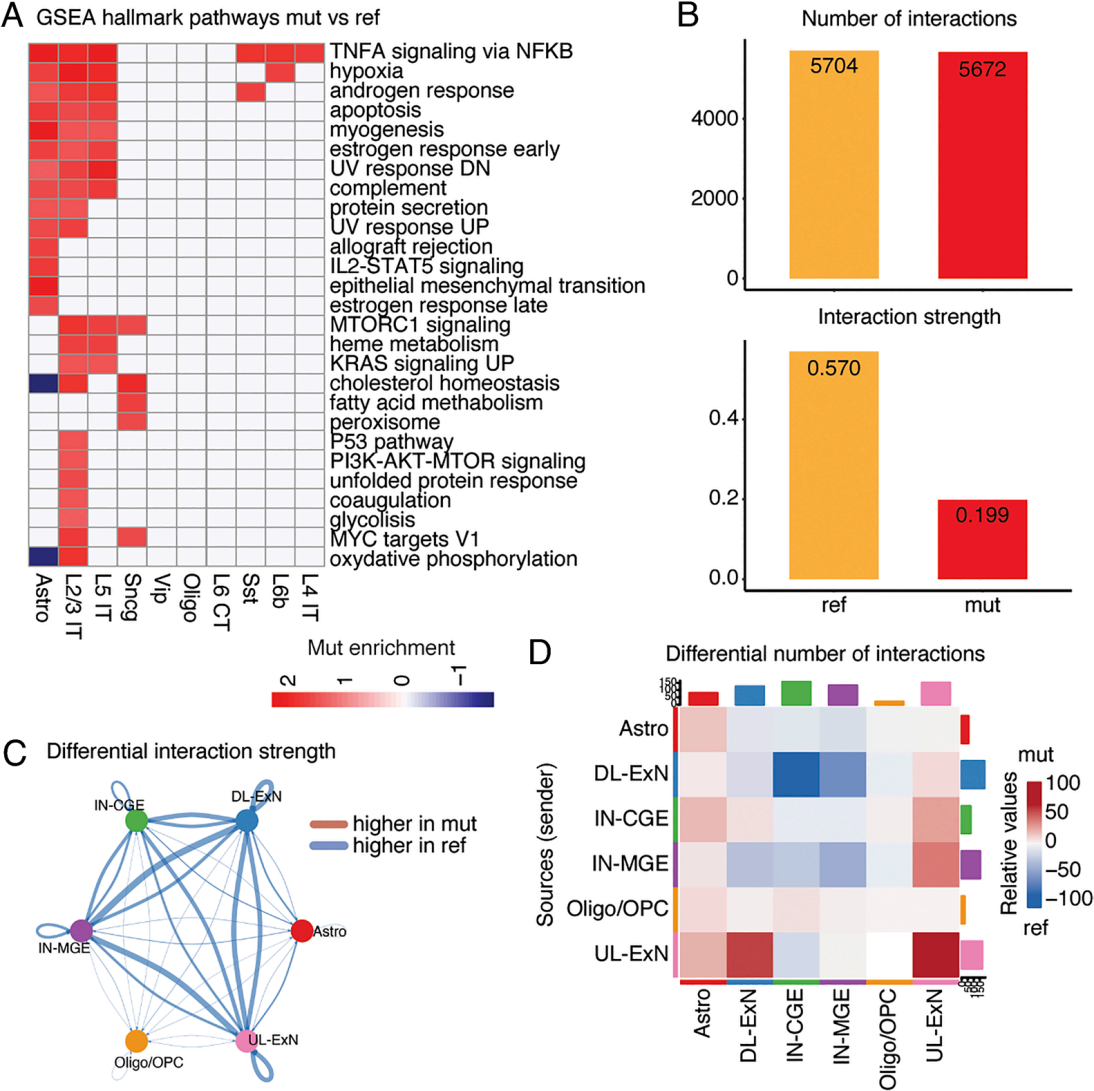
Genotype-associated transcriptional alterations. (*A*) Heatmap showing Molecular Signatures Database Hallmark pathways (33, 34) enriched in mut compared to ref per cell type, obtained with fgsea (35). High-sensitivity calls were used for this analysis. (*B*) Bar plots displaying overall number of interactions and interaction strength predicted by CellChat (46) in mut vs. ref nuclei. (*C*) Circos plot where edge thickness represents the GO-TEN mut vs. ref differential interaction strength calculated with CellChat. (*D*) Heatmap displaying differential number of interactions between cell types in mut vs. ref nuclei obtained with CellChat. Sender cell types are reported on the y-axis.

Genotype-informed analysis of transcription revealed dramatic differences between variant-carrying and non-variant-carrying cells within the lesion. DGE and GSEA (35) using the fifty Hallmark Pathways (33, 34) on GO-TEN genotyped ref and mut nuclei in our dataset (*SI Appendix*, Fig. S16 and Datasets S16 and S17), using data from nuclei genotyped with the high-sensitivity approach—but restricted to cell types carrying the variant in the high-specificity dataset—showed upregulation of PI3K-mTOR and related pathways, such as MTORC1, KRAS, PI3K-AKT-MTOR, and MYC targets V1 pathways, in mut cells despite the global downregulation of these pathways in the genotype-uninformed sn-RNAseq (Fig. 6*A*, *SI Appendix*, Fig. S17*A*, Datasets S16 and S17). These results suggest that mut cells in mosaic FCD2 display cell-autonomous pathological hyperactivation of PI3K-mTOR and related pathways, while surrounding and more abundant non-variant-carrying cells may compensate by non-cell-autonomously downregulating such pathways, resulting in global downregulation when compared to control brains. We also detected upregulation of pathways indicative of inflammation and immune activation such as TNFA signaling via NFKB and IL2-STAT5 signaling (Fig. 6*A*). Similar results were obtained when performing GSEA on ResolveOME data (*SI Appendix*, Fig. S17*B* and Dataset S18). Finally, mut cells showed upregulation of several terms associated with synapses and epilepsy (*SI Appendix*, Fig. S17 *C* and *D*).

GO-TEN genotyped mut nuclei showed no clear differences in the global numbers of interactions compared to ref, but a marked decrease in interaction strength between mut cells (Fig. 6*B*) using CellChat (46). This was particularly true for interactions between mut neuronal subtypes, both excitatory and inhibitory (Fig. 6*C*). When looking at numbers of interactions, CellChat revealed reduced number of interactions between mut excitatory neurons (DL-ExN) and interneurons (IN-CGE and IN-MGE) and increased number of interactions among mut excitatory neurons (DL-ExN and UL-ExN) compared to ref (Fig. 6*D* and *SI Appendix*, Fig. S18*A*). Signaling pathways potentially underlying such changes included affecting pathways such as NRXN, ADGRL, PTPR, NEGR, NRG, and glutamate in neurons (*SI Appendix*, Fig. S18 *B* and *C* and Dataset S19). To conclude, this analysis suggests that while changes in connectivity may be more severe between variant-carrying cells compared to non-variant-carrying cells in the mosaic FCD2 brains, disrupted connectivity likely involves all cells irrespective of their genotype, overall contributing to circuit hyperexcitability.

## Discussion

Using high-throughput single-cell transcriptomics of mosaic FCD2 spectrum brain lesions harboring somatic activating PI3K-mTOR variants, we showed that the vast majority of mutant cells have preserved cell identities comparable to controls. While this finding may appear contradictory to the findings of a previously published study (15), joint analysis of our data and the raw data from Chung et al. was consistent in that it did not identify any transcriptionally distinct abnormal cell clusters in FCD2. This finding was replicated even when separately analyzing high VAF samples, where one might expect abundant mutant dysplastic cells. Furthermore, disease and control groups had similar global cell type compositions. Thus, somatic variants in FCD2 do not appear to create new, disease-associated cell identities, suggesting that DNs and BCs, in spite of their distinctive histopathological appearance, are either exceedingly rare as a proportion of the cell population, and thus not well captured by our workflow, lost during sample prep due to their unique properties, filtered out during data processing, or have similar transcriptional identities to their nondysplastic counterparts. This is also consistent with other reports of normal-appearing neurons in FCD2 carrying pathogenic somatic variants (2). In future studies, more targeted technologies such as laser capture or DEPArray (61) may be used to isolate dysplastic cells from FCD2 tissue and study the specific transcriptional and posttranscriptional alterations that may underlie functional differences among dysplastic and nondysplastic cells.

Astrocytes, microglia-PVM, OPCs, and endothelial cells in FCD2 appeared enriched in lesions compared to controls. While this analysis is subject to differential sampling, they may indicate gliosis and microglial activation in the FCD2-associated dysplastic cortex, which is supported by DGE and GSEA. These results support prior reports of inflammation in FCD2 tissue (24, 62), but do not clearly establish whether such changes are a contributor to or a consequence of epileptic seizures. Future studies may investigate roles of microglia in FCD2 pathophysiology in greater depth, since these could represent a promising therapeutic target. For example, inhibition of one of the significantly upregulated signaling pathways in FCD, JAK-STAT3, was recently shown to have profound seizure suppressive properties in a mouse model of focal epilepsy (63).

Genotype-informed DGE and GSEA identified variant-specific alterations in cellular metabolism, biosynthesis, and growth-related pathways common to multiple cell types in FCD2. While PI3K-mTOR and related pathways were globally downregulated in neurons in FCD2 compared to controls overall, variant-carrying neurons in FCD2 tissue showed the expected upregulation of these pathways compared to non-variant-carrying counterparts. This is indicative of a complex dynamic where cells in the affected mosaic tissue all exhibit dysregulated signaling and dysfunction irrespective of their genotype, and helps resolve the paradox of why FCD2 lesions show hypometabolism on clinical PET imaging (28–30), despite overactivation of PI3K-mTOR signaling caused by the somatic variants. It also illustrates the critical importance of genotype-informed RNAseq analysis as prior bulk RNA-seq studies (31, 32) were unable to identify these complementary changes due to lower statistical power and also lack of cell type–resolved analyses. Indeed, pseudobulk analysis of our RNA-seq data did not identify significant differential regulation of PI3K-mTOR pathway, despite a very significant signal when comparing neurons alone, showing the power of snRNA-seq when looking at the mechanisms of mosaic neurologic diseases in tissue.

Interrogation of epilepsy-associated pathways and gene lists, together with CellChat analysis, strongly suggested global alterations in synapse formation and function and cell–cell connectivity in FCD2, with a global decrease in the stability of neuronal connectivity in FCD2 compared to control brains, which is even more evident in variant-carrying compared to non-variant-carrying cells. We found this dysfunction to be particularly significant between excitatory neurons and interneurons, potentially contributing to hyperexcitability. Although functional studies are needed to definitively test these hypotheses, our data suggest that mosaic variants lead to epilepsy via broad changes in neuronal circuits rather than cell-autonomous hyperexcitability alone.

GO-TEN and ResolveOME cell-type-informed genotyping of *PIK3CA* and *MTOR* somatic variants in seven patients found that variant-carrying cells map to well-differentiated cell types, further suggesting that the major impact of FCD2-related variants is not creating new cell identities, but instead altering the transcriptomic states and signaling of well-differentiated cells. FCD2 somatic variants are rarely found outside the brain and most studies to date support restriction of pathogenic somatic variants in FCD2 to the cerebral cortex (2, 3), although exceptions to this have been reported for chromosome 1q gains (60). Our GO-TEN and ResolveOME data consistently support restriction of FCD2-associated variants to cells derived from a neuroectodermal progenitor, supporting the commonly held belief that most such variants arise in cortical neural progenitor cells. This finding is inconsistent with a recent study reporting variant-carrying mesoderm-derived microglia and endothelial cells in FCD2 (54). Although this apparent discrepancy deserves further exploration, one possible explanation may be the differences among the genotyping approaches. Due to a much higher number of on-target reads, GO-TEN has the benefit of a strict statistical framework based on base quality, UMI count, and nucleus-level filtering to achieve high genotyping accuracy. Furthermore, ResolveOME allows direct profiling of genomic DNA, which is even more accurate than cDNA-based methods.

The focal nature of cortical findings in FCD2 suggests a simple model that the FCD2-associated variants first arise in dorsal forebrain progenitors that produce radially migrating neurons and glial cells. Co-occurrence of somatic variants in excitatory and inhibitory neurons confirms prior reports of variant-carrying interneurons in FCD2 (64), and is consistent with the claim of a shared dorsal progenitor for these two neuronal types (13, 57–59), though we cannot definitively exclude that some FCD2 mutations occur at earlier stages.

Our study is limited by the low sensitivity of GO-TEN, which in its current form can genotype ~21% of the sequenced cells in the high-sensitivity mode, with sensitivity that may be even lower depending on gene expression levels. This drawback leads to reduced sensitivity of analyses that aim at detecting genotype-associated gene expression changes. A second limitation is the technically demanding nature of the GO-TEN technology that results in incomplete genotyping of all the available alleles. Finally, our regression analysis reveals that certain cell types are preferentially genotyped for a given gene, perhaps due to differential isoform usage although future studies are needed to investigate this hypothesis. Although ResolveOME addresses some of these limitations by performing genotyping on the amplified DNA and by genotyping both alleles, it is significantly more expensive and has lower throughput.

Despite these limitations, our study highlights the importance of investigating both cell-autonomous and non-cell-autonomous mechanisms of somatic variants in mosaic FCD2, showing that both aspects are likely involved in disease pathophysiology. Furthermore, we illustrate the disruption of intercellular communication in neurons that likely plays a key role in epileptogenesis and chronic epilepsy. Our single-cell genotyping strategy focused on GoF variants in two well-established FCD2 genes, *PIK3CA* and *MTOR*, could be applied at a larger scale in future studies to address the potentially different impact of specific genes and variants in FCD, and help generate more effective, targeted pharmacological treatments.

## Methods

### Patient Cohort and Human Tissue.

This study was approved by the Institutional Review Board (IRB) of Boston Children’s Hospital (05-05-76R and 09-02-0043). Subjects were identified and evaluated in a clinical setting, and surgical brain specimens were collected for research after obtaining written informed consent. Deidentified patient samples were also obtained from surgical resections following written informed consent from Thomas Jefferson University Hospital (IRB approval 98.0550), Cleveland Clinic Epilepsy Biospecimen Bank (IRB approval 12-1000), Melbourne Brain Centre (Austin Health IRB approval 02961) and European Epilepsy Brain Bank (University Hospital Erlangen approval 193_18B).

Neurotypical control postmortem prefrontal cortex and occipital cortex tissue was obtained from a 15-y-old female (UMB4638) and a 42-y-old female (UMB4643), respectively. Neurotypical control postmortem temporal neocortical tissue was obtained from a 48-y-old male (UMB1570) and a 48-y-old female (UMB1739). None of the individuals had a history of neurologic disease. Tissue was obtained from the NIH NeuroBioBank at the University of Maryland Brain and Tissue Bank (UMBTB). As approved by the University of Maryland IRB, research on these deidentified specimens and data was performed at Boston Children’s Hospital.

### Single-Nuclei RNA-Sequencing.

Fresh-frozen postoperative and postmortem brain tissues stored at −80 °C were dissected in a cryostat chamber kept at constant −20 °C to obtain samples of ~20 to 30 mg. When possible, a homogeneous mix of gray and white matter was dissected. Nuclei preparations were performed as previously described (65). For some preparations, an intermediate step consisting of DAPI staining and fluorescence-activated nuclear sorting (FANS) was performed (*SI Appendix*, Fig. S1*B*). Nuclei sorting was performed on a FACS Aria II cell sorter equipped with BD FACSDiva software, selecting all DAPI-positive nuclei (SSC-A and FSC-A gate boundaries were set at 10^3^ and 30, respectively). snRNA-seq was performed using the 10X Genomics Chromium Next GEM Single Cell 3′ Reagent Kit v3.0 and v3.1. Around 30,000 nuclei were used to load the 10X Chromium, and were processed the same day for gel-bead in emulsion (GEM) generation, barcoding, and cDNA amplification, following manufacturer instructions. Libraries were sequenced (paired-end single indexing) on an Illumina NovaSeq6000 targeting ~50,000 read-pairs per single-nucleus.

### Single-Nucleus RNA-Sequencing analysis.

Alignment and gene expression quantification of snRNA-seq data generated for this manuscript and from Chung et al. (15), was performed with the 10X CellRanger pipeline (v8.0.1) based on the GRCh38 reference genome using all default parameters and with introns included (--include-introns flag). CellBender (v0.3.0) (66) with all default parameters was used to adjust raw counts based on automatic detection of likely contaminant reads (20). Likely doublets were detected and removed with scrublet (v0.2.3) (19).

Seurat (v5.1.0) (26) and Harmony (v1.2.0) (16) were used for data normalization, integration, clustering, and annotation (*SI Appendix*).

### Differential Cell Type Composition Analysis.

Differences in cell type composition between cases and controls were assessed using a chi-squared proportion test (base R *chisq.test*) and using propeller (v1.2.0) (23) (*P*-values are reported in Dataset S2).

### Differential Gene Expression and Pathway Enrichment Analysis.

Differential gene expression analysis was performed using Seurat’s default *FindMarkers* function and two different tests for differential expression run independently: 1) Wilcoxon rank sum test, and 2) MAST (67). Only genes expressed in at least 10% of nuclei within a particular comparison were considered (min.pct = 0.10) and no log2 fold-change threshold was imposed (logfc.threshold = 0). Default parameters were used otherwise. Gene set enrichment analysis was performed with fgsea (v1.28.0) (35), with genes ranked in descending order by average log2 fold-change. A Benjamini–Hochberg correction was used to control for multiple hypothesis testing. Relevant ontologies were obtained from MSigDB (34) (https://www.gsea-msigdb.org/gsea/msigdb), DisGenNet (43) (https://www.disgenet.com/), Macnee et al. (42), SynGO (41) (https://www.syngoportal.org/) and SFARI Gene (44) (https://gene.sfari.org/).

### Microglial State Analysis.

A one-sided hypergeometric test was applied between DEGs (average log-2 fold-change > 0.5 and adj. *P* < 0.05) upregulated in microglia and microglial state genes (36). Multiple hypothesis testing correction was performed using Benjamini–Hochberg.

### CellChat Analysis.

CellChat (v2.1.2) (46) was used to identify changes in cell–cell communication networks following standard practices (*SI Appendix*).

### GO-TEN and ResolveOME Single Nucleus Genotyping.

GO-TEN and ResolveOME genotyping protocols and bioinformatic analyses are described in details in *SI Appendix*.

## Supplementary Material

Appendix 01 (PDF)

Dataset S01 (XLSX)

Dataset S02 (XLSX)

Dataset S03 (XLSX)

Dataset S04 (XLSX)

Dataset S05 (XLSX)

Dataset S06 (XLSX)

Dataset S07 (XLSX)

Dataset S08 (XLSX)

Dataset S09 (XLSX)

Dataset S10 (XLSX)

Dataset S11 (XLSX)

Dataset S12 (XLSX)

Dataset S13 (XLSX)

Dataset S14 (XLSX)

Dataset S15 (XLSX)

Dataset S16 (XLSX)

Dataset S17 (XLSX)

Dataset S18 (XLSX)

Dataset S19 (XLSX)

## Data Availability

Code for creating and analyzing data utilized in this manuscript are available on Github at https://github.com/MayaTalukdar/single-cell-analysis-of-fcd (68). Newly generated deidentified human single-nucleus RNA-seq data are available at dbGaP under the accession number phs004124.v1.p1 and are available upon request if access is granted at https://www.ncbi.nlm.nih.gov/projects/gap/cgi-bin/study.cgi?study_id=phs004124.v1.p1 (11).
