# Supplementary material for: Cell-type-informed genotyping of mosaic focal epilepsies reveals cell-autonomous and non-cell-autonomous disease-associated transcriptional programs
Source: Proc Natl Acad Sci U S A. 2025 Jul 17;122(29):e2509622122. doi: 10.1073/pnas.2509622122 (PMC12305027; doi:10.1073/pnas.2509622122)
Supplement: Supplementary file 1 — Appendix 01 (PDF) [file pnas.2509622122.sapp.pdf]

PNAS

Supporting Information for

Cell-type-informed genotyping of mosaic focal epilepsies reveals cell-autonomous and non-cell-autonomous disease-associated transcriptional programs

Sara Bizzotto[1,2,3,4,*,#], Maya Talukdar[1,2,3,5,*], Edward A. Stronge[1,2,3,5,*], Rosita B. Ramirez[6], Yingxi Yang[1,2,3,6], August Yue Huang[1,2,3], Qiwen Hu[7], Yingping Hou[6], Norma K. Hylton[1,2,3,5], Benjamin Finander[1,2,3], Ashton Tillett[6], Zinan Zhou[1,2,3], Brian H. Chhouk[1,2,3], Alissa M. D'Gama[2,8,9], Edward Yang[10], Timothy E. Green[11], David C. Reutens[12], Saul A. Mullen[11], Ingrid E. Scheffer[11], Michael S. Hildebrand[11,13], Russell J. Buono[14], Ingmar Blümcke[15,16], Annapurna H. Poduri[3,8], Sattar Khoshkhoo[1,3,6,†,#], Christopher A. Walsh[1,2,3,17,18,†,#]

[1] Division of Genetics and Genomics, Manton Center for Orphan Disease Research, Boston, MA 02115

[2] Departments of Pediatrics, Boston Children's Hospital, Harvard Medical School, Boston, MA 02115

[3] Broad Institute of Massachusetts Institute of Technology and Harvard, Cambridge, MA 02115

[4] Université Paris Cité, Imagine Institute, Team Somatic Mosaicism in Neurodevelopment and Disease, Paris 75015, France

[5] Harvard/Massachusetts Institute of Technology MD-PhD Program, Harvard Medical School, Boston, MA 02115

[6] Department of Neurology, Brigham and Women's Hospital, Harvard Medical School, Boston, MA 02115

[7] Department of Biomedical Informatics, Harvard Medical School, Boston, MA 02115

[8] Epilepsy Genetics Program, Division of Epilepsy and Neurophysiology, Department of Neurology, Boston Children's Hospital, Harvard Medical School, Boston, MA 02115

[9] Division of Newborn Medicine, Department of Pediatrics, Boston Children's Hospital, Harvard Medical School, Boston, MA 02115

[10] Department of Radiology, Boston Children's Hospital, Boston, MA 02115

[11] Department of Medicine (Austin Health), University of Melbourne, Heidelberg, VIC 3084, Australia

[12] Centre for Advanced Imaging, The University of Queensland and Royal Brisbane and Women's Hospital, Herston, QLD 4067, Australia

[13] Neuroscience Group, Murdoch Children's Research Institute, Parkville, VIC 3052, Australia

[14] Department of Biomedical Sciences, Cooper Medical School of Rowan University, Camden, NJ 08103

[15] Department of Neuropathology, University Hospitals Erlangen, Erlangen 91054, Germany

[16] Epilepsy Center, Cleveland Clinic, Cleveland, OH 44106

[17] Allen Discovery Center for Human Brain Evolution, Boston Children's Hospital, Harvard Medical School, Boston, MA 02115

[18] Howard Hughes Medical Institute, Boston, MA 02115

* First authors

† Senior authors

# Correspondence to: christopher.walsh@childrens.harvard.edu; skhoshkhoo@bwh.harvard.edu; sara.bizzotto@inserm.fr

This PDF file includes:

Table S1
Supporting text
Extended methods
Figures S1 to S18
Legends for Datasets S1 to S19
SI References

Other supporting materials for this manuscript include the following:

Datasets S1 to S19

Table S1. Sample cohort						
**Sample ID**	**Case ID**	**Brain region**	**Sample type**	**Diagnosis**	**Variant**	**VAF (%)**
E274	E274	L-CC	FF surgical	HME	*PIK3CA* p.E542K (1)	25.8
E241	E241	L-CC	FF surgical	HME	*PIK3CA* p.E545K (1)	23.5
E348	E348	R-CC	FF surgical	HME	*PIK3CA* p.E545K (1)	16.4
E174-1	E174	R-CC	FF surgical	HME	*PIK3CA* p.E542K (2)	15.9-17.4
E174-2	E174	R-CC	FF surgical	HME	*PIK3CA* p.E542K	13.9
E254	E254	R-CC	FF surgical	HME	*MTOR* p.C1483R (1)	23.7
E276	E276	-	FF surgical	HME	*MTOR* p.C1483Y (1)	11.7
FC5801	FC5801	L-H	FF surgical	FCD2B	*MTOR* p.C1483R (2)	10.0-10.6
E286	E286	R-CC	FF surgical	FCD2B	*MTOR* p.S2215F (1)	5.8
E364	E364	L-FC	FF surgical	FCD2	*MTOR* p.T1977K (1)	4.7
E244	E244	L-FC	FF surgical	FCD2	*MTOR* p.T1977R (1)	2.8-4.7
E316	E316	R-POTC	FF surgical	FCD2	*MTOR* p.S2215Y (1)	4.5
FC5501	FC5501	L-FPC	FF surgical	FCD2B	*MTOR* p.L1460P (2)	2.3-2.6
FC6401	FC6401	L-FC	FF surgical	FCD2B	*TSC1* p.Q55X (2)	5.1-6.7
E203	E203	L-FTC	FF surgical	FCD2A	1q dup (*AKT3*) (1)	NA
FC10801	FC10801	L-FC	FF surgical	FCD2B	*DEPDC5* p.Gln1501*	germline
HE3601	HE3601	R-CC	FF surgical	HME	-	-
FC5901	FC5901	L-FC	FF surgical	FCD2B	-	-
EP39801	EP39801	L-H	FF surgical	HME	PIK3CA p. E542K	17.7
EP41101	EP41101	L-H	FF surgical	HME	*PIK3CA* p.E545K	20.9
HME_4688*	HME_4688	CC	FF surgical	HME	*PIK3CA* p.E545K	25.1
HME_6593*	HME_6593	CC	FF surgical	HME	*PIK3CA* p.H1047R	13.1
TSC_4258*	TSC_4258	CC	FF surgical	TSC	NA	NA
TSC_4259*	TSC_4259	CC	FF surgical	TSC	NA	NA
UMB4638-S1**	UMB4638	L-PFC	FF postmortem	NT ctrl	-	-
UMB4638-S2	UMB4638	L-OC-V1	FF postmortem	NT ctrl	-	-
UMB4638-S3	UMB4638	L-OC-V1	FF postmortem	NT ctrl	-	-
UMB4638-S4	UMB4638	L-OC-V1	FF postmortem	NT ctrl	-	-
UMB4638-S5	UMB4638	L-OC-V2	FF postmortem	NT ctrl	-	-
UMB4638-S6	UMB4638	L-OC-V2	FF postmortem	NT ctrl	-	-
UMB4643-S1**	UMB4643	L-PFC	FF postmortem	NT ctrl	-	-

UMB4643-S2	UMB4643	L-OC-V1	FF postmortem	NT ctrl	-	-
UMB4643-S3	UMB4643	L-OC-V1	FF postmortem	NT ctrl	-	-
UMB4643-S4	UMB4643	L-OC-V2	FF postmortem	NT ctrl	-	-
UMB4643-S5	UMB4643	L-OC-V2	FF postmortem	NT ctrl	-	-
NIH_Ctrl_1570	UMB1570	TC	FF postmortem	NT ctrl	-	-
NIH_Ctrl_1739	UMB1739	TC	FF postmortem	NT ctrl	-	-
M1C***	NA	PMC-PC	FF postmortem	NT ctrl	-	-
MTG***	NA	MTG	FF postmortem	NT ctrl	-	-
S1C***	NA	PSC-PoCG	FF postmortem	NT ctrl	-	-
A46***	NA	MFG	FF postmortem	NT ctrl	-	-
A32***	NA	DD-MFC-RG	FF postmortem	NT ctrl	-	-
A14***	NA	MOfC-RG	FF postmortem	NT ctrl	-	-
A19***	NA	SOG	FF postmortem	NT ctrl	-	-
CTRL_8352_8353*	CTRL_8352_8353	CC	FF postmortem	NT ctrl	-	-
TLE36	TLE36	TC	FF surgical	mTLE ctrl	-	-
TLE6	TLE6	TC	FF surgical	mTLE ctrl	-	-
TLE7	TLE7	TC	FF surgical	mTLE ctrl	-	-
FC10101	FC10101	TC	FF surgical	mTLE ctrl	-	-

Abbreviations: L-CC, left cerebral cortex; R-CC, right cerebral cortex; L-FPC, left frontoparietal cortex; L-H, left hemisphere; L-FC, left frontal cortex; R-POTC, right parieto-occipital temporal cortex; L-FTC, left frontotemporal cortex; L-PFC, left prefrontal cortex; L-OC-V1, left occipital cortex primary visual; L-OC-V2, left occipital cortex secondary visual; TC, temporal cortex; PMC-PC, primary motor cortex precentral gyrus; MTG, middle temporal gyrus; PSC-PoCG, primary somatosensory cortex postcentral gyrus; MFG, middle frontal gyrus; MFC-RG, dorsal division of medial frontal cortex rostral gyrus; MOfC-RG, medial orbitofrontal cortex gyrus rectus; SOG, superior occipital gyrus; FF, fresh-frozen; NT, neurotypical; mTLE, mesial temporal lobe epilepsy; NA, not available; * samples from Chung et al. (3); **, samples previously published by us in Ganz et al. (4); ***, samples from the BRAIN Initiative Cell Census Network Human Brain Cell Atlas v1.0 (5).

Supporting text

Absence of clusters corresponding to dysplastic cells in FCD2 snRNA-seq data

Several possible explanations for the absence of clusters containing dysplastic cells (DNs and BCs) in snRNA-seq data include that 1) integration of case and control datasets forces the dysplastic cells into clusters shared among the FCD and control samples; 2) the rarity of these cell types may require even larger sample size to be captured; 3) BCs are frequently multinucleated and hence may be filtered by doublet removal methods.

To assess the first hypothesis, we performed integration and unsupervised clustering in FCD2 samples only (without controls) using three different methods (Seurat Canonical Correlation Analysis (CCA), Seurat Reciprocal Principle Component Analysis (RPCA), and Harmony) (6, 7). On each integrated FCD2 object, we performed cluster annotation using Azimuth (7) and the Allen motor cortex cell atlas (8) as reference, and verified these annotations by examining expression patterns of known brain cell type marker genes (Fig. S4 and Fig. S5A). All the clusters in the FCD2 object mapped with high confidence (prediction scores > 0.6) to a control cell type and again no novel or unmapped clusters were identified, which was independently replicated across all three integration methods (Fig. S5B-E).

In order to assess the second hypothesis about the rarity of dysplastic cells, we performed a similar analysis to the one mentioned above, focusing on four cases carrying a pathogenic variant with high VAF (E174, VAF = 13.9 – 17.4%; E348, VAF = 16.4%; E274, VAF = 25.8%; E254, VAF = 23.7%) that we analyzed individually without data integration. We performed this analysis with the assumption that VAF generally correlates with dysplastic cell density in FCD2 and therefore if dysplastic cells adopted unique cell identities, we would have the highest chance at identifying them in these samples. Despite the high proportion of variant-carrying cells in these samples, similar to the integrated analyses, we observed high reference cell type prediction scores, and found no evidence of a unique dysplastic cell cluster (Fig. S7).

Finally, we assessed the third hypothesis by analyzing our snRNA-seq data without filtering out predicted putative doublets (Fig. S8A-C). As would be expected for true doublets, we found that the doublet ratio in each sample was positively correlated with cell count (Fig. S8D; Pearson's coefficient $r = 0.63$, $p = 0.00618$). However, we did not observe significant correlation between doublet ratio and VAF of carried variants in individual samples (Fig. S8E; Pearson's coefficient $r = 0.35$, $p = 0.245$). This analysis suggests that BCs are not enriched in doublets that were detected in our data and removed bioinformatically.

Extended methods

Single-nucleus RNA-sequencing data processing

We analyzed snRNA-sequencing data from three sources (Table S1): 1) data generated for this manuscript, 2) previously published data from Chung et al. (3), and 3) previously published data from Siletti et al. (5) Data generated from this manuscript and from Chung et al. includes both controls and FCD2 cases. Data from Siletti et al. consists solely of neurotypical brain controls sequenced as part of the BRAIN Initiative Cell Census Network. We specifically focused on samples from seven cortical areas of interest, matched to the areas sampled in our data and from Chung et al.: 1) primary motor cortex (M1C); 2) middle temporal gyrus (MTG); 3) primary somatosensory cortex (S1C); 4) middle frontal gyrus (A46); 5) dorsal division of MFC (A32); 6) medial orbitofrontal cortex (A14); and 7) superior occipital gyrus - areas 19 and MT (A19). Given the large number of nuclei captured in the Siletti et al., study, we downsampled each cortical region to 20,000 nuclei proportional to the cell type composition in the original sample, using cell types defined by Siletti et al., in their publication associated metadata (5).

Seurat (v5.1.0) (7) was used as follows: we combined count matrices from all samples generated for this manuscript, samples utilized from Chung et al. (3), and downsampled (as described above) count matrices from Siletti et al (5). High-quality nuclei were extracted based on the following criteria: 1) percent mitochondrial gene expression < 5%; 2) percent ribosomal gene expression < 5%; 3) number of expressed genes (nFeature_RNA) > 300; and 4) number of transcripts (nCount_RNA) > 300 (Fig. S1C). snRNA-seq was then normalized (*NormalizeData)* and scaled (*ScaleData)*, with regression of the covariates of percent mitochondrial gene expression, percent ribosomal gene expression, nFeature_RNA, and nCount_RNA. Dimensionality reduction was performed using Principal Component Analysis (PCA, *RunPCA)*, and the top 30 principal components (PCs) were used for downstream analysis based on visual inspection of an elbow plot of the proportion of variance captured by each PC. All samples were integrated (*IntegrateLayers)* using Harmony (v1.2.0) (6) utilizing all default parameters and operating on the PC embeddings, as Harmony has

been found to outperform other single-cell integration methods in benchmarking studies (9). UMAP (*RunUMAP)* was used for dimensionality reduction and visualization of this integrated Harmony embedding, and unsupervised clustering was performed on this integrated Harmony embedding using Louvain's algorithm (*FindNeighbors* and *FindClusters)* with multiple resolutions (0.6, 1.2) to ensure capture of granular cell types.

Annotation was performed by first generating likely cell type assignments for each unsupervised cluster using the Seurat reference-based single-cell analysis tool Azimuth (7) and a reference dataset of the adult human motor cortex (8). Cell type annotations were then further refined by examining the expression of canonical brain marker genes (5, 10) (Fig. S2).

Cell-chat analysis

To compare inferred communication networks between different conditions, we individual CellChat (11) objects were generated by subsetting the original Seurat v5 objects based on conditions, as described by the CellChat developers here: https://htmlpreview.github.io/?https://github.com/jinworks/CellChat/blob/master/tutorial/CellChat-vignette.html and here https://htmlpreview.github.io/?https://github.com/jinworks/CellChat/blob/master/tutorial/Comparison_analysis_of_multiple_datasets.html/. To ensure sufficient power to determine differences between conditions, neuronal types were grouped into broader cell class categories: MGE interneurons (Pvalb, Sst), CGE interneurons (Vip, Sncg, Lamp5), upper-layer excitatory neurons (L2/3 IT), and deeper-layer excitatory neurons (L4 IT, L5 IT, L5 ET, L5/6 NP, L6 CT, L6 IT, and L6 IT Car3). The curated CellChatDB.human database was used to infer all cell-cell interactions based on known receptor-ligand pairing data. For each object individually, gene expression data was projected onto CellChat's protein-protein interaction network (*identifyOverExpressedGenes* and *identifyOverExpressedInteractions*), inferred and filtered the overall cell-cell communication network (*commuteCommunProb, filterCommunication, computeCommunProbPathway*), ending with the final aggregated cell-cell communication network *(aggregateNet)*. CellChat objects corresponding to different conditions were thus merged (*mergeCellChat*) and compared the total number of interactions and interaction strength (*compareInteractions*, *netVisual_diffInteractions, netVisual_heatmap)*. To understand the signaling pathways driving these differential interactions, the information flow for each signaling pathway was compared between conditions (*rankNet*).

GO-TEN single-nucleus genotyping

Excess full-length cDNA from 10X Genomics Next GEM Single Cell 3′ GEM Kit v3.1 workflow was used as input for the GO-TEN protocol. 10 ng of excess cDNA after the amplification step was used to perform hemi-nested long-range PCR to capture the target variant as well as the unique 10X barcode and UMI. The PrimeSTAR GXL DNA Polymerase was used, following the manufacturer's manual, given optimal performance at the desired amplicon length. The long-range PCR product was then used for long-read sequencing library preparation using the ONT Kit12 chemistry. The final library was sequenced on one MinION Flow Cell.

For genotyping of known variant sites, MinION reads were aligned with MiniMap2 (v2.24) (12) to the GRCh38 human reference genome, and reads that did not align to the targeted locus of interest were discarded. Each read was parsed based on the expected structure of the read (i.e. known primer sequences, 10X cell barcodes, unique molecular identifiers (UMIs), and poly-A tail) to identify the putative allele (i.e. reference or expected variant) present at the known variant site. A small portion of reads were identified as containing neither the reference nor the expected variant allele; these reads were discarded as they likely represent sequencing errors. Reads that did not contain a 10X CBC that was present in the final processed snRNA-seq dataset were also discarded. 179,385 (FC5501), 94,407 (FC5801), and 5,641 (e174) long reads were sequenced that met the aforementioned criteria.

Genotyping data was used to address two major biological questions: 1) identification of variant cell types, and 2) transcriptomic comparison of mut and ref nuclei. The first analysis (i.e. variant cell type identification) is inherently more sensitive to false positive calls than the second analysis (i.e. transcriptomic comparison of mut and ref nuclei), as a small number of false positive genotyping calls can lead to incorrect identification of variant cell types and thus the developmental timing of our somatic variants of interest (i.e. whether the variant arose pre- or post-gastrulation). In contrast, as nuclei are pooled together when comparing mut versus ref nuclei, such transcriptomic comparisons are less sensitive to the presence of a small percentage of false positive nuclei, but they require a sufficiently large number of nuclei to be able to achieve adequate statistical power for downstream analysis. Given these two complementary questions and their distinct technical requirements, we implemented two downstream genotyping approaches: 1) a "strict" high-

specificity genotyping approach for which reads that did not contain a 10X UMI identified in our snRNA-seq data corresponding to the gene of interest were further filtered out; 2) a “loose” high-sensitivity genotyping approach, in which we did not apply the latter filter.

By applying the high-specificity approach, 109,151 (FC5501), 37,108 (FC5801), and 2,040 (e174) long reads were retained (Datasets S9 to S13). Importantly, UMIs corresponding to these reads did not correspond to 10X UMIs otherwise identified in snRNA-seq library that were off-target for *PIK3CA* or *MTOR*, nor did they represent 10X UMIs with sequencing errors, as we allowed an edit distance of up to two nucleotides between ONT and 10X UMIs. The vast majority of these reads likely still represent high-quality reads: for example, this approach discards reads whose 10X CBC was retained in snRNA-seq library but whose specific UMI was filtered out. Moreover, as GO-TEN directly enriches for transcripts from target genes of interest, some ONT reads will be derived from rare, on-target transcripts that are not captured in snRNA-seq library. Still, we reasoned that such an approach minimizes the chance of incorporating reads that have an unexpected structure or are off-target for the locus of interest (although the vast majority of these reads will be filtered by additional, aforementioned filtering criteria) and is thus sufficiently stringent for variant cell type identification. Transcriptomic comparison of mut versus ref nuclei was restricted to variant cell types determined by the high-specificity calling.

UMI and read support metrics are listed below for both genotyping approaches and are comparable.

Genotype	# of Nuclei With 1 UMI Support	# of Nuclei With 2 UMI Support	# of Nuclei With >2 UMI Support	# of UMIs with 1 Read Support	# of UMIs with 2 Read Support	# of UMIs with >2 Read Support
High sensitivity approach						
Reference	2536	721	625	10542	559	639
Mutant	587	370	945	13635	836	1028
High specificity approach						
Reference	153	5	8	1847	255	516
Mutant	28	3	8	1151	141	236

Final genotype calls per nucleus were generated with scMosaicHunter (13, 14). scMosaicHunter performs genotyping by integrating both UMI and nucleus-level information, rather than genotyping based on arbitrarily defined cutoffs for read support or mutant read fraction per nuclei. Specifically, a genotype is first determined for each UMI (i.e. ref or mut) based on aggregating multiple reads originating from the same UMI. These UMI genotypes are then aggregated together to call final genotypes per nucleus. In each step, both base quality and the number of reads/UMIs per UMI/nucleus, respectively, are considered. Moreover, the variant cell fraction estimated from bulk DNA sequencing is additionally considered as the prior probability in the second step that an individual nucleus is mut as compared to ref.

With the high-sensitivity approach, 1,798 nuclei were genotyped as mut and 3,636 nuclei were genotyped as ref. With the high-specificity approach, 39 nuclei were genotyped as mut and 166 as ref.

GO-TEN genotyping efficiency analysis

We sought to understand the discrepancy between mosaic fractions obtained from bulk DNA sequencing and those obtained with GO-TEN by analyzing covariates that may affect genotyping efficiency. To identify covariates associated with genotyping efficiency (i.e. the probability a nucleus was genotyped confidently, regardless of whether it was identified as ref or mut), we fit the following logistic regression model (R *stats* package v4.3.1 *glm* function) across all nuclei successfully genotyped based on both our genotyping approaches (Fig. S13C), as described above: nucleusWasGenotyped (binary outcome; 1 = was genotyped and 0 = was not genotyped) ~ expression of target gene + cell type.

We found that the expression level of a gene in a given cell positively correlated with the probability of genotyping, which is expected since GO-TEN genotypes cDNA molecules. Surprisingly, however, we also found that some cell types had increased or decreased probability of being genotyped for a specific gene variant (Fig. S13C). This represents an area of future investigation; however, we speculate that it may be a consequence of the expression of specific transcript isoforms that may be more or less efficiently captured by GO-TEN. Thus, there are at least two potential contributors to the inconsistency between the mosaic fractions derived from bulk VAFs and the mosaic fractions obtained with GO-TEN: 1) because GO-TEN genotypes cDNA, the expression level of a given gene is expected to be broadly correlated with genotyping probability, which is consistent with an underestimation of the mosaic fraction obtained for *PIK3CA* (5.1 – 7.6% GO-TEN versus 27.8 – 34.8% bulk DNA; GTEX (15) cortex expression level 4.49 TPM) and an overestimation of the mosaic fraction obtained for *MTOR* (25 – 52.2% GO-TEN versus 20 – 21.6% bulk DNA for FC5801, 21.6 – 43.5% GO-TEN versus 4.6 – 5.2% bulk DNA for FC5501; GTEX cortex expression level 13.96 TPM); 2) the probability of genotyping varies among different cell types.

<u>ResolveOME single nucleus RNA sequencing and DNA genotyping</u>

Fresh-frozen postoperative brain tissues stored at -80 °C were dissected in a cryostat chamber kept at constant -20 °C to obtain samples of ~5-10 mg. After dissection, samples were immediately transferred in ice chilled nuclear lysis buffer (10 mM Tris-HCl PH8, 0.25 M Sucrose, 5 mM $MgCl_2$, 25 mM KCl, pH 8, 1 uM DTT, and 0.2 U/µl RNase inhibitor) and lysed using a dounce homogenizer. The homogenate was filtered through a 40 µm cell strainer. Homogenates were mixed 1:1 with a 42% Iodixanol solution (0.075 M Sucrose, 5 mM $MgCl_2$, 25 mM KCl, 10 mM Tris-HCl pH 8, 42% iodixanol w/v) and then layered on top of 25% Iodixanol cushion buffer (0.146 M Sucrose, 10 mM $MgCl_2$, 48 mM KCl, 19 mM Tris-HCl pH 8, 25% iodixanol w/v) and centrifuged for 15 minutes at 8,000 rcf. Pellets containing nuclei were resuspended in 500 µl ice-cold resuspension buffer (1X BSA in PBS supplemented with 5 mM $MgCl_2$ and 0.2 U/µl RNase inhibitor). Nuclei suspensions were centrifuged at 500 rcf and 4 °C for 5 minutes, and pellets re-suspended in 100 µl immunostaining buffer plus DAPI for FANS. Nuclei sorting was performed on a FACS Aria II cell sorter equipped with BD FACSDiva software, selecting all DAPI-positive nuclei. Single nuclei were sorted into 384 well plates (1 nucleus per well) on top of 1uL of Cell Buffer (Bioskryb Genomics, Durham, NC) and the plates were flash-frozen and sored at -80 °C. For single-nucleus DNA amplification and RNA-sequencing, two different versions of a commercially available kit ResolveOME (Bioskryb Genomics) were used.

Sample E174 was processed using 96X ResolveOME v1 and samples E274, E286, EP39801, and EP41101 were processed using 384X ResolveOME v2. Plates containing sorted nuclei were thawed on ice and underwent DNA amplification and RNA library preparation following the manufacturer's protocols with minor modifications as follows: 1) for the sample processed with ResolveOME v1, the reagent volumes were reduced to 1/3 of the amounts recommended in the protocol to optimize for 384-well format; 2) for RNA library preparation, the cDNA input amounts varied from 10 ng to 20 ng depending on cDNA yield; 3) for nuclei with low cDNA concentration, vacuum concentration was performed to meet the ResolveOME RNA library prep input requirements. The snRNA-seq libraries were pooled and sequenced at 300 reads/cycle on a NextSeq 1000 Illumina device. On average 37296 raw sequencing reads per cell were generated.

<u>Digital droplet PCR on ResolveOME amplified DNA</u>

To determine genotype, digital droplet PCR (ddPCR) was performed on the amplified DNA for each cell using commercially available Taqman primer/probe mixes chosen based on the specific variant present in each sample:

**Sample**	**Gene**	**Variant Position**	**Base Change**	**Protein Change**	**Taqman Probe**
E174	PIK3CA	chr3:179218294	G>A	p. E542K	C_169027163_10
E274	PIK3CA	chr3:179218294	G>A	p. E542K	C_169027163_10
E286	MTOR	chr1:11124516	G>A	p. S2215F	C_362039351_10
EP39801	PIK3CA	chr3:179218294	G>A	p. E542K	C_169027163_10

EP41101	PIK3CA	chr3:179218303	G>A	p. E545K	C_150852487_1 0

ddPCR was performed according to the guidelines published in Droplet Digital PCR Applications Guide (Bio-Rad). 10-50 ng of ResolveOME-amplified DNA, TaqMan primer/probe mix (1X), ddPCR Supermix for Probes (1X, Bio-Rad), and nuclease-free H_2O were mixed for a total volume of 20 µL. Droplets were generated using the QX200 droplet generator (Bio-Rad), and PCR was performed with 25 cycles of amplification. Droplets were evaluated using the QX200 droplet reader (Bio-Rad).

ddPCR data were analyzed using QuantSoft (Bio-Rad). VAFs were calculated based on the total number of ref and alt alleles. To increase our confidence in genotype assignment we required >200 genotyped droplets/nucleus. Nuclei with alt VAF > 5% were labeled as “het-mut” and nuclei with alt VAF < 1% were labeled as “ref-hom.” Nuclei that didn’t meet these requirements were labeled as “unknown” and excluded from downstream analyses.

<u>ResolveOME snRNA-seq analysis</u>

Raw sequencing reads were aligned to the human reference genome (GRCh38) using STAR v2.7.11b (16), followed by the generation of gene count matrices with featureCounts v2.0.8 (17), as previously described (18). Quality control was performed to exclude low-quality cells based on the following criteria: 1) fewer than 100 expressed genes, 2) over 50% ribosomal gene counts, or 3) over 50% mitochondrial gene counts. The filtered count data were normalized using the *NormalizeData* function in Seurat v5 (7). For cell-type annotation, each nucleus was assigned a predicted cell type by mapping to the FCD2-only snRNA-seq reference dataset using Seurat’s *FindTransferAnchors* and *TransferData* functions, with the first 30 principal components. Cell type identities were determined based on the highest proportion of transferred labels and validated using canonical marker genes.

To identify cell-type-specific and overall transcriptional changes between het-mut and ref-hom nuclei defined by ddPCR, we filtered the nuclei with stricter quality criteria: nuclei with at least 300 expressed genes, no more than 10% ribosomal gene counts, and no more than 20% mitochondrial gene counts. Only the cell types with both het-mut and ref-hom nuclei were included for cell-type-specific and overall differential gene expression analysis using Seurat’s *FindMarkers* function. Genes were filtered to include those expressed in a minimum of 5% of nuclei (min.pct = 0.05) and exhibiting a log2 fold-change greater than 0.1 (logfc.threshold = 0.1). GSEA was conducted using the fgsea package (19), ranking genes by descending average log2 fold-change. Due to the limited number of nuclei, no multiple testing corrections were applied.

Figures S1 to S18

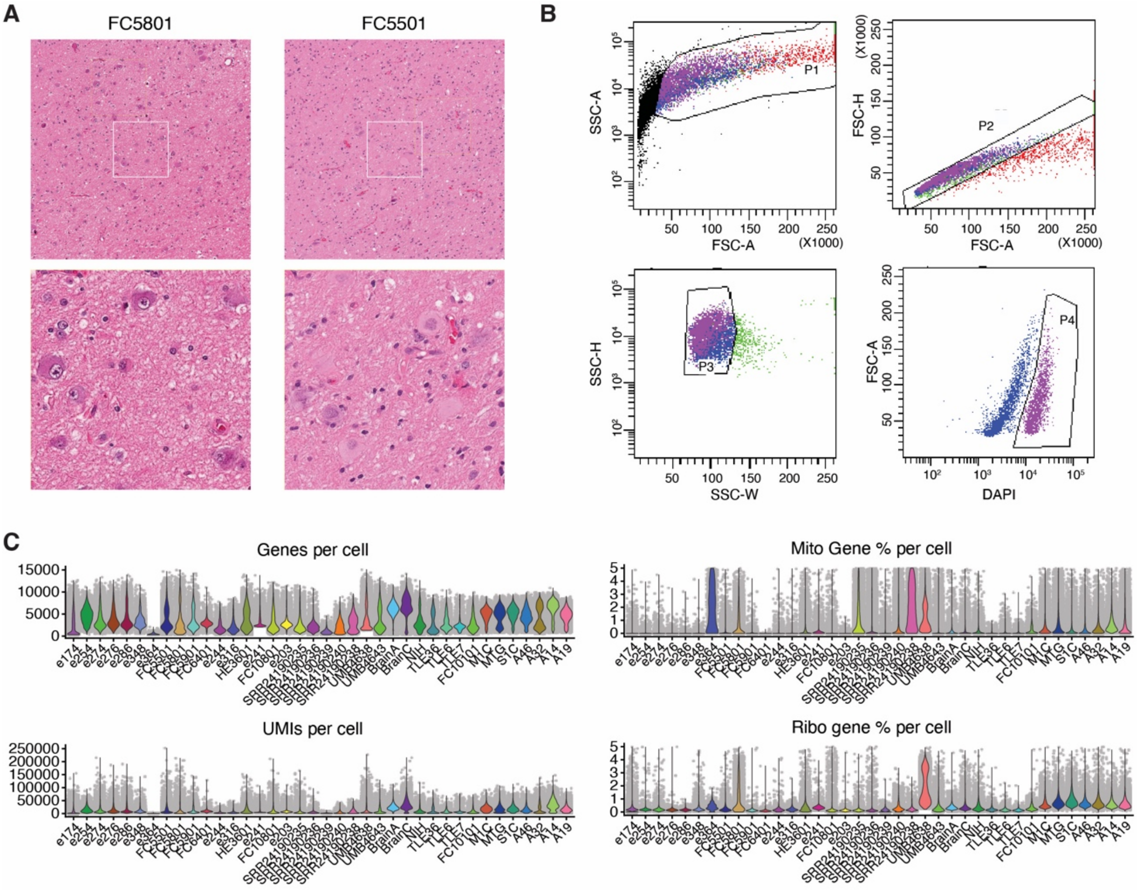

Fig. S1. (**A**) Representative hematoxylin and eosin (H&E) histopathology staining from two patients in our cohort, showing the presence of dysmorphic neurons and balloon cells. (**B**) Representative images of the gating strategy performed for DAPI fluorescent activated nuclear sorting upstream of single-nuclei RNA-sequencing. (**C**) Distribution of snRNA-seq quality parameters for each sample included in this study.

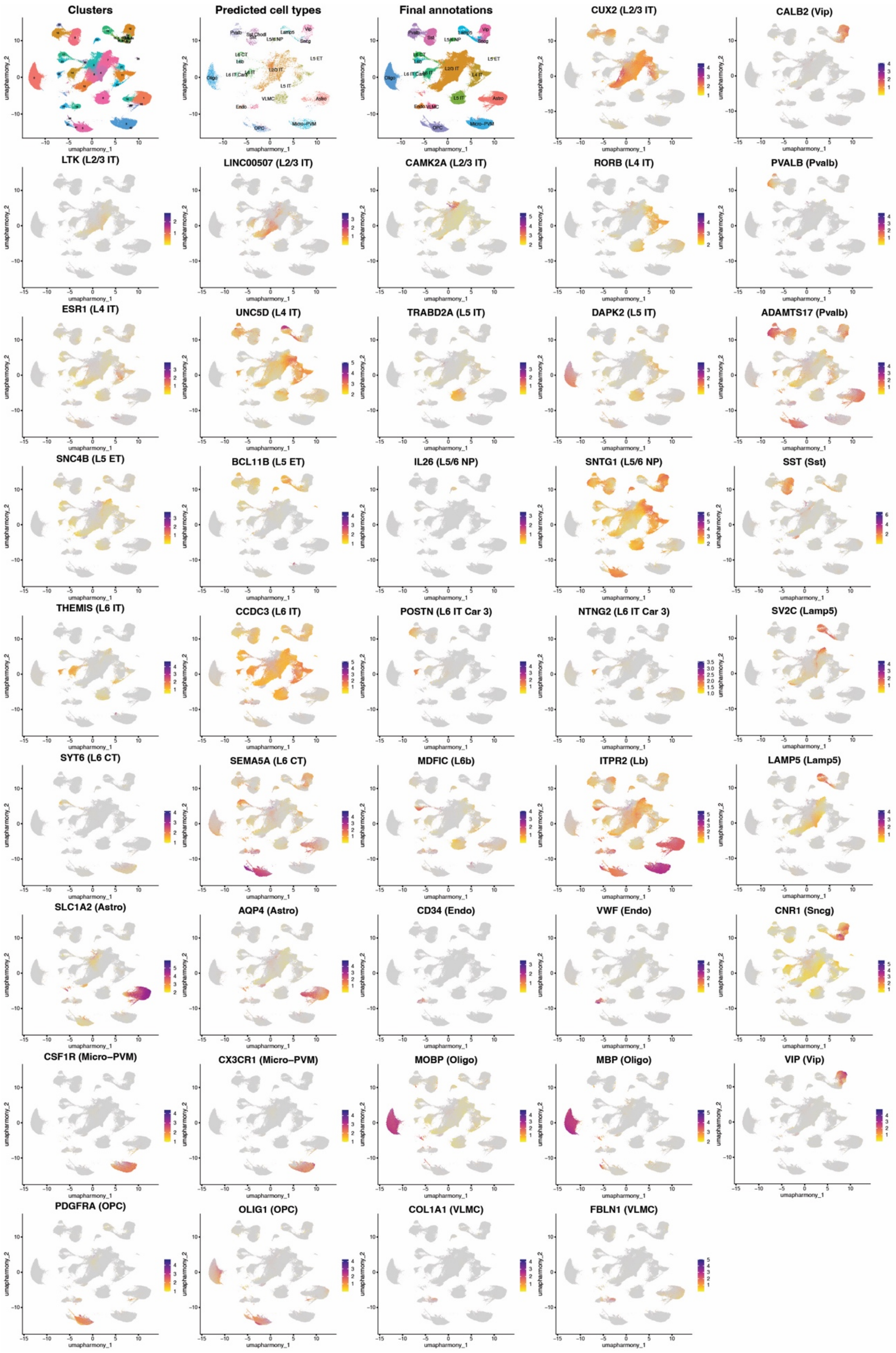
Clusters
Predicted cell types
Final annotations
CUX2 (L2/3 IT)
CALB2 (Vip)
LTK (L2/3 IT)
LINC00507 (L2/3 IT)
CAMK2A (L2/3 IT)
RORB (L4 IT)
PVALB (Pvalb)
ESR1 (L4 IT)
UNC5D (L4 IT)
TRABD2A (L5 IT)
DAPK2 (L5 IT)
ADAMTS17 (Pvalb)
SNC4B (L5 ET)
BCL11B (L5 ET)
IL26 (L5/6 NP)
SNTG1 (L5/6 NP)
SST (Sst)
THEMIS (L6 IT)
CCDC3 (L6 IT)
POSTN (L6 IT Car 3)
NTNG2 (L6 IT Car 3)
SV2C (Lamp5)
SYT6 (L6 CT)
SEMA5A (L6 CT)
MDFIC (L6b)
ITPR2 (Lb)
LAMP5 (Lamp5)
SLC1A2 (Astro)
AQP4 (Astro)
CD34 (Endo)
VWF (Endo)
CNR1 (Sncg)
CSF1R (Micro–PVM)
CX3CR1 (Micro–PVM)
MOBP (Oligo)
MBP (Oligo)
VIP (Vip)
PDGFRA (OPC)
OLIG1 (OPC)
COL1A1 (VLMC)
FBLN1 (VLMC)
umapharmony_1
umapharmony_2

Fig. S2. Data integration and cell type annotation including all case and control nuclei. Data integration was performed with Harmony (6). Uniform Manifold Approximation and Projection (UMAP) dimensionality reduction plots displaying 1) unsupervised clusters, 2) predicted cell types obtained by Azimuth (7) using a reference snRNA-seq atlas of the human motor cortex as reference (8), and 3) the final annotation obtained by combining predicted cell types with expression patterns of canonical brain cell type marker genes (5, 10). Cell type predictions were run on a downsampled object that had all the clusters proportionally represented. Feature plots display canonical brain cell type marker genes.

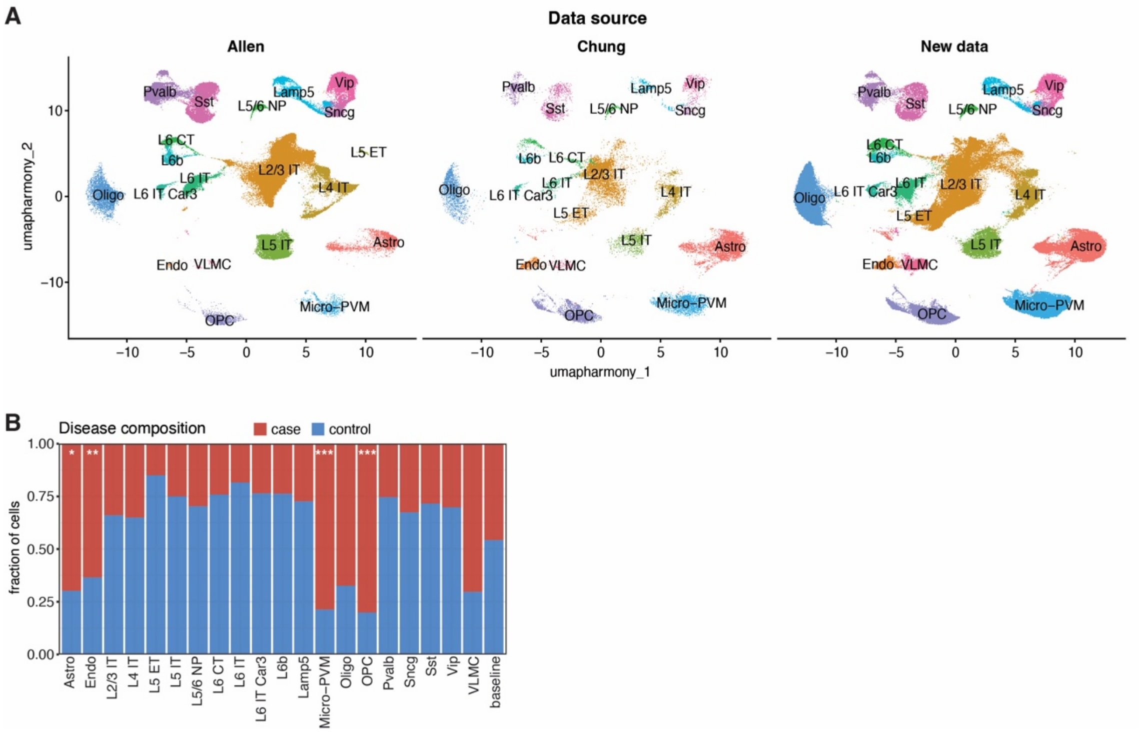

Fig. S3. (**A**) UMAPs displaying successful integration of datasets coming from different sources using Harmony (v1.2.0). (**B**) Contribution of disease and control samples to different annotated cell types showing significant enrichment of microglia-PVMs, OPCs, astrocytes and endothelial cells in disease compared to control. Statistical analyses were done using propeller (20) (see Methods and Dataset S2). ***, adj. $p < 0.001$; **, adj. $p < 0.01$; *, adj. $p < 0.05$.

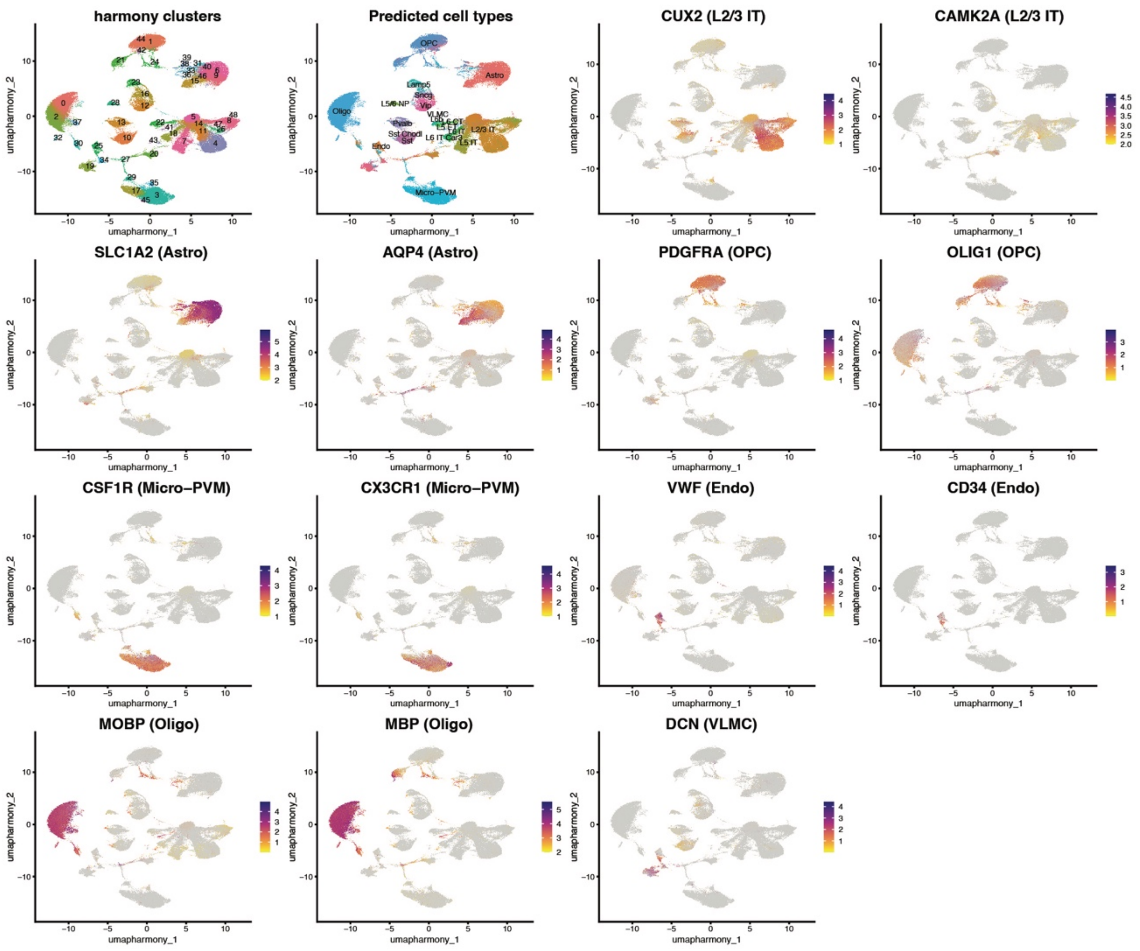

Fig. S4. UMAP plots showing only FCD2 samples integrated with Harmony with cell type predictions by Azimuth using a reference snRNA-seq atlas of the human motor cortex as reference. Feature plots show canonical brain cell type marker gene expression, confirming fidelity of cell type annotation.

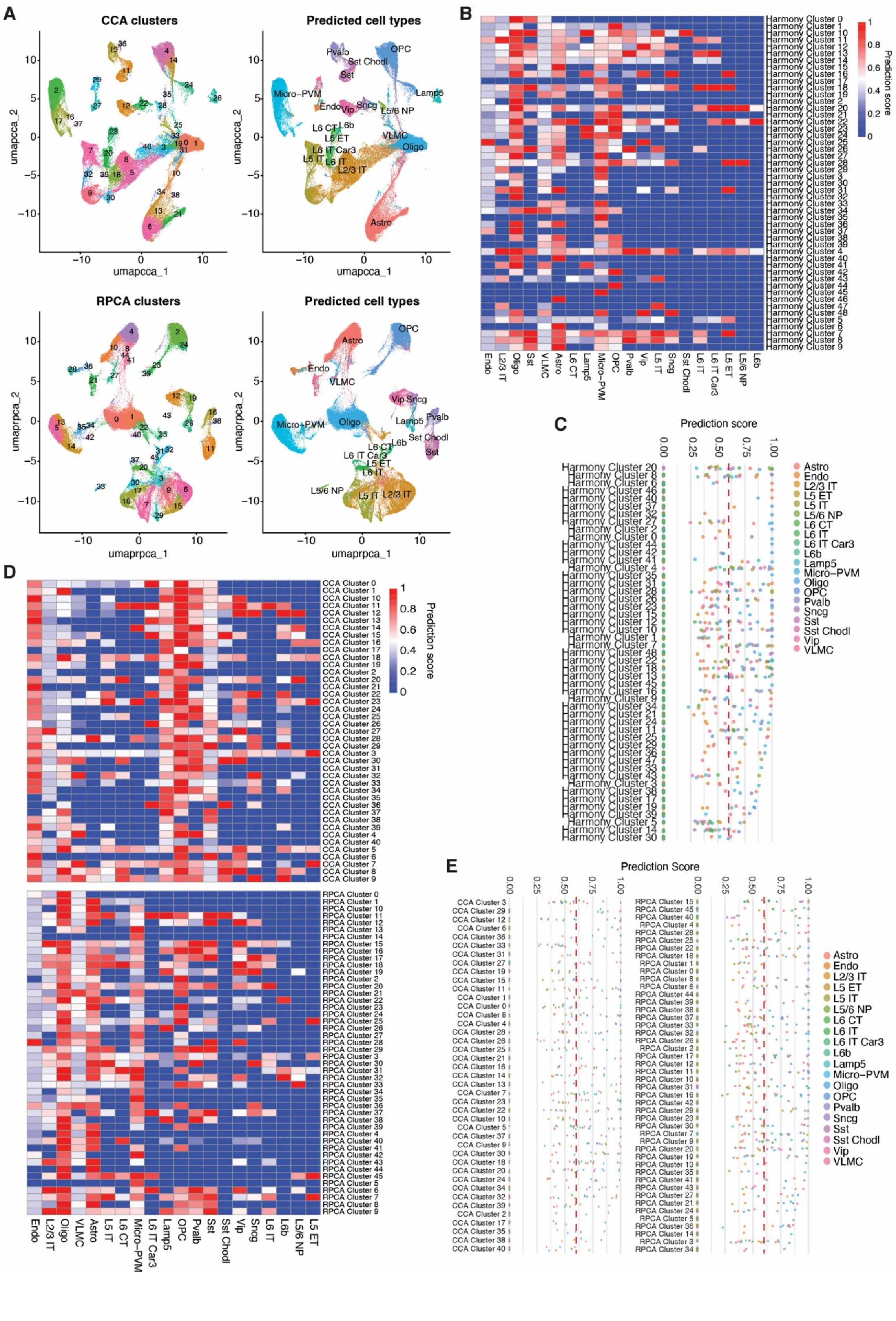

A
CCA clusters
Predicted cell types
umapcca_1
umapcca_2
RPCA clusters
umaprpca_1
umaprpca_2
Micro−PVM
Endo
Vip
Sncg
L5/6 NP
L6 CT
L6b
L5 ET
L6 IT Car3
L5 IT
L6 IT
L2/3 IT
VLMC
Oligo
OPC
Pvalb
Sst Chodl
Sst
Lamp5
Astro
B
Prediction score
C
Prediction score
D
Prediction score
E
Prediction Score
Astro
Endo
L2/3 IT
L5 ET
L5 IT
L5/6 NP
L6 CT
L6 IT
L6 IT Car3
L6b
Lamp5
Micro−PVM
Oligo
OPC
Pvalb
Sncg
Sst
Sst Chodl
Vip
VLMC

Fig. S5. (**A**) UMAPs displaying unsupervised clusters and predicted cell types using Seurat CCA and RPCA integration (7) for disease samples only. Cell types were predicted using Azimuth and a reference snRNA-seq atlas of the human motor cortex. (**B**) Heatmap displaying Azimuth cell type prediction scores for each cluster in the Harmony-integrated FCD2 data. (**C**) Dot plot showing high (> 0.6) Azimuth cell type prediction scores for each unsupervised cluster in the Harmony-integrated FCD2 data. (**D**) Heatmaps displaying Azimuth cell type prediction scores for each cluster in the CCA and RPCA integrated disease data. (**E**) Dot plots showing high (> 0.6) Azimuth cell type prediction scores for each cluster in the CCA and RPCA integrated disease data.

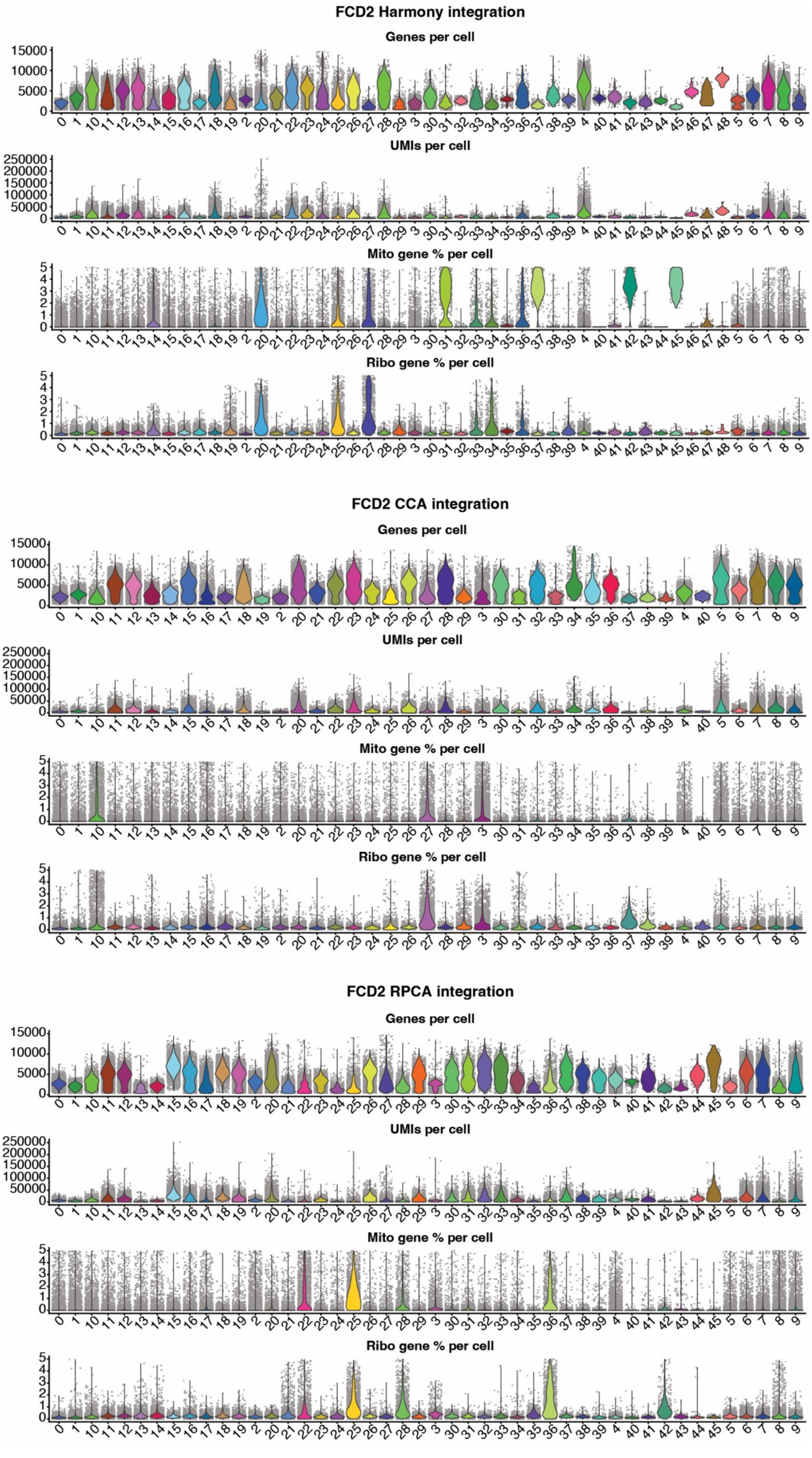
FCD2 Harmony integration
Genes per cell
UMIs per cell
Mito gene % per cell
Ribo gene % per cell
FCD2 CCA integration
Genes per cell
UMIs per cell
Mito gene % per cell
Ribo gene % per cell
FCD2 RPCA integration
Genes per cell
UMIs per cell
Mito gene % per cell
Ribo gene % per cell

Fig. S6. Per-cluster QC parameters for FCD2 sample integration with Harmony, Seurat CCA and Seurat RPCA (7).

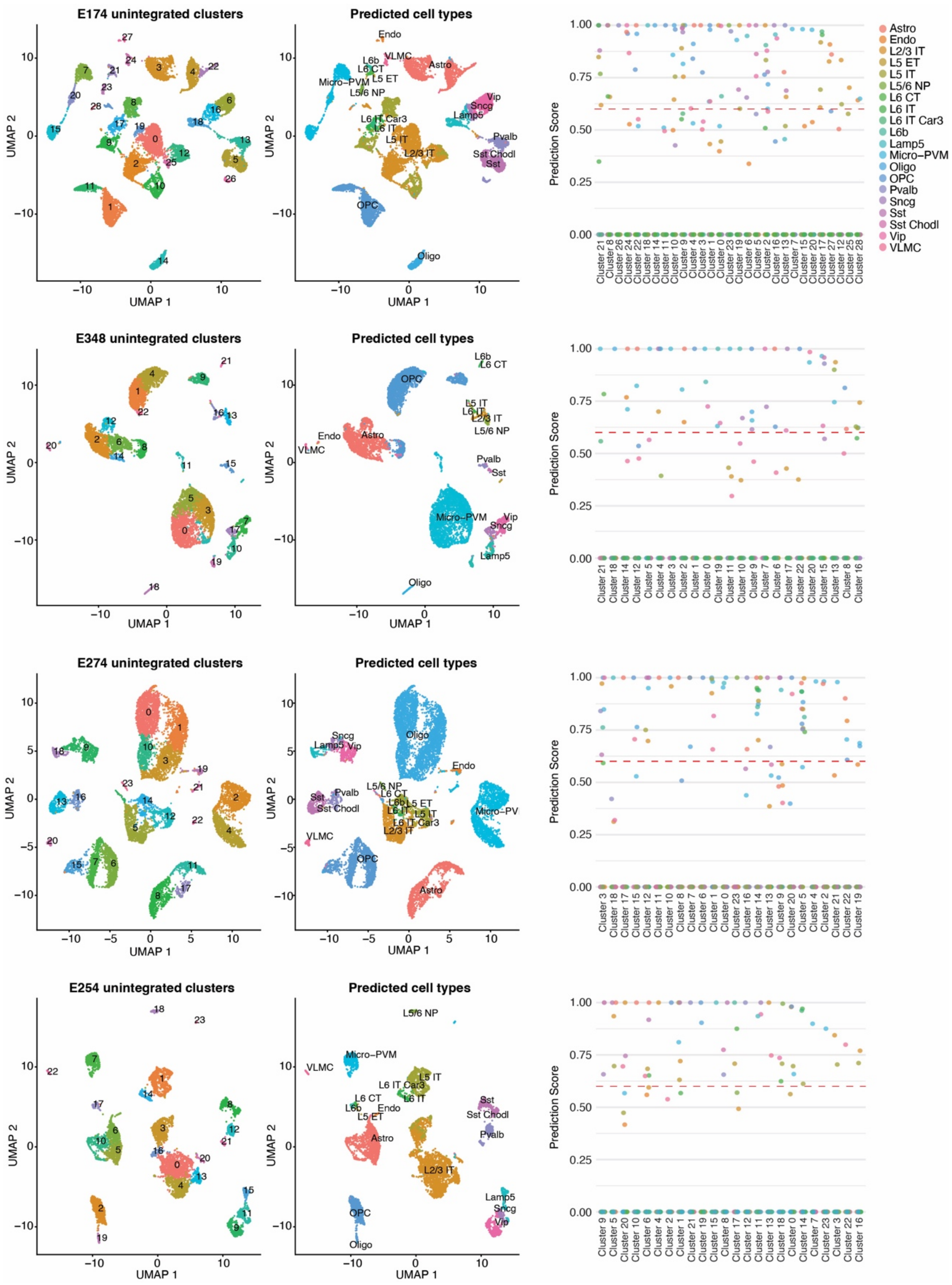

Fig. S7. UMAPs displaying unsupervised clusters and predicted cell types in four high variant allele frequency (VAF) disease samples. Cell type predictions were obtained using Azimuth and a

reference snRNA-seq atlas of the human motor cortex. High prediction scores (> 0.6) for each cluster are displayed in the dot plots on the right.

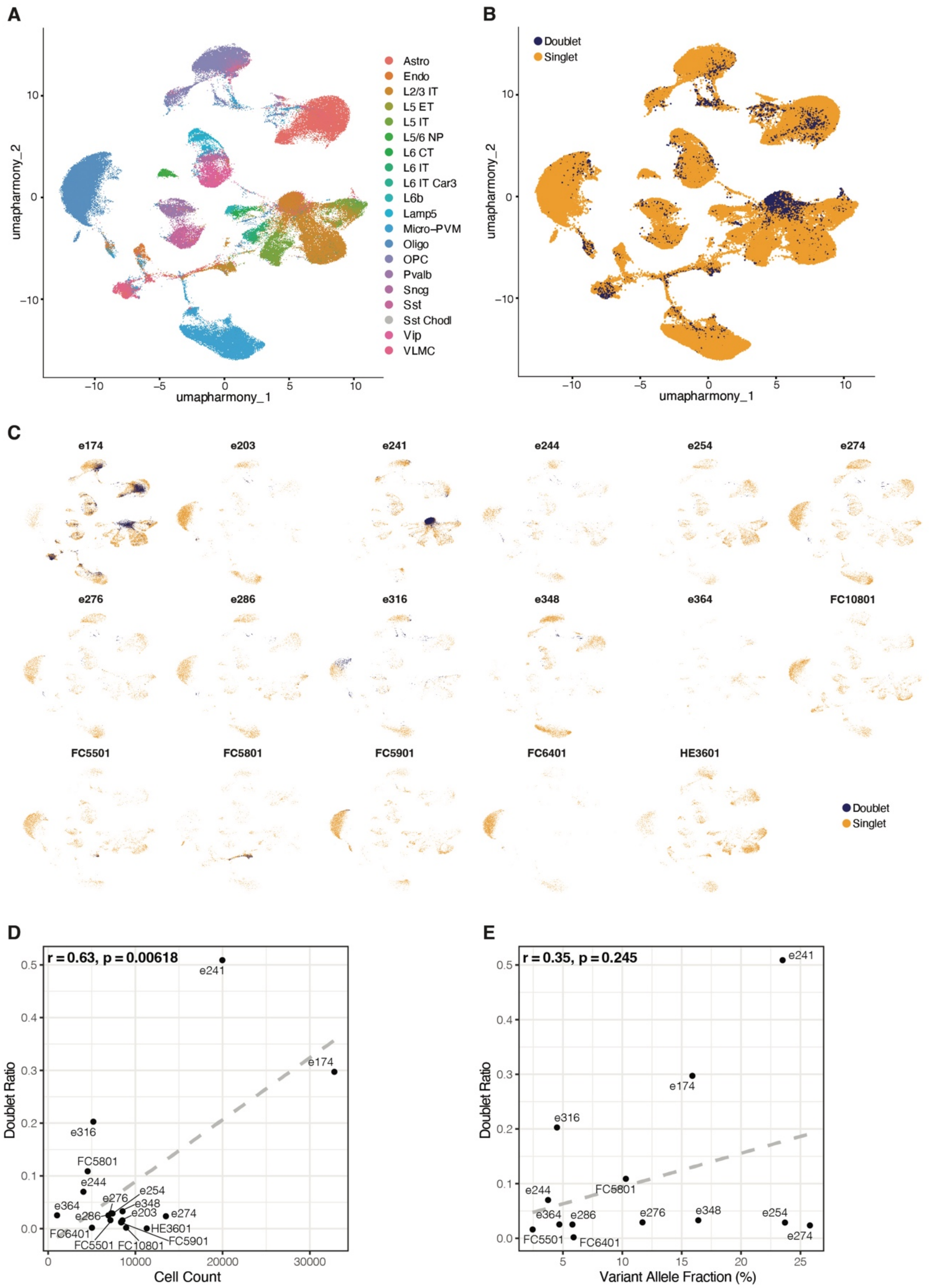
A
B
C
D
E
Astro
Endo
L2/3 IT
L5 ET
L5 IT
L5/6 NP
L6 CT
L6 IT
L6 IT Car3
L6b
Lamp5
Micro–PVM
Oligo
OPC
Pvalb
Sncg
Sst
Sst Chodl
Vip
VLMC
umapharmony_1
umapharmony_2
Doublet
Singlet
e174
e203
e241
e244
e254
e274
e276
e286
e316
e348
e364
FC10801
FC5501
FC5801
FC5901
FC6401
HE3601
r = 0.63, p = 0.00618
Doublet Ratio
Cell Count
r = 0.35, p = 0.245
Variant Allele Fraction (%)

Fig. S8. (**A**) UMAP displaying integrated (Harmony) case samples without performing doublet removal. Cell types were predicted with Azimuth using a reference snRNA-seq atlas of the human motor cortex. (**B**) UMAP displaying singlets and doublets as predicted by *scrublet (21)*. (**C**) Predicted singlet and doublet distribution per case sample. (**D**) Significant positive correlation between per-sample doublet ratio and nuclei count. (**E**) No significant correlation between per-sample doublet ratio and VAF. *r*, Pearson's correlation coefficient.

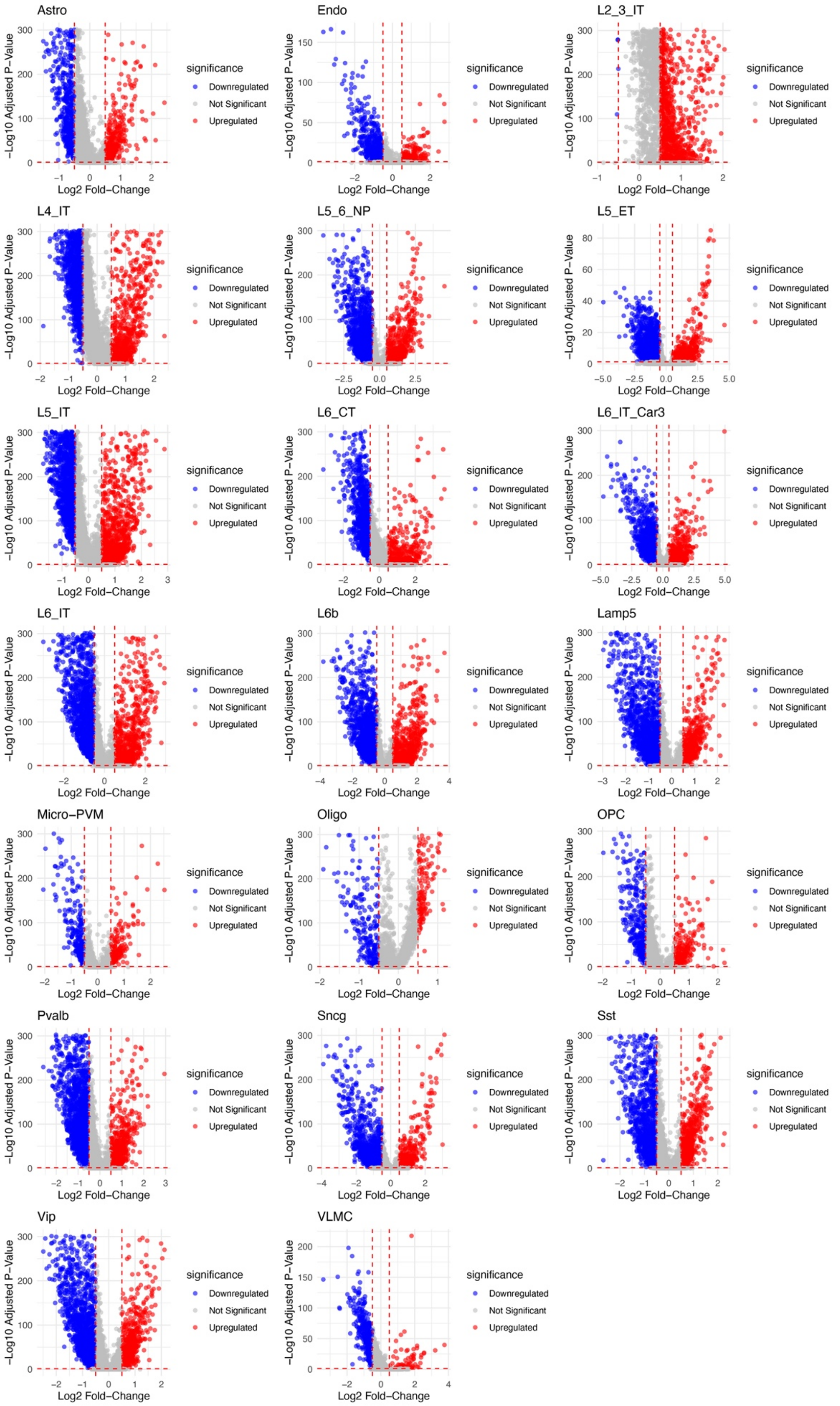
Astro
Endo
L2_3_IT
L4_IT
L5_6_NP
L5_ET
L5_IT
L6_CT
L6_IT_Car3
L6_IT
L6b
Lamp5
Micro−PVM
Oligo
OPC
Pvalb
Sncg
Sst
Vip
VLMC
−Log10 Adjusted P−Value
Log2 Fold−Change
significance
Downregulated
Not Significant
Upregulated

Fig. S9. Volcano plots displaying cell-type-specific differentially expressed genes (DEGs) between case and control samples. Differential expression analysis was done with Seurat *FindMarkers* using a Wilcoxon rank sum test with a Benajmini-Hochberg correction for multiple hypothesis testing. DEGs with an average log-2 fold-change with absolute value > 0.5 and adjusted *p*-values < 0.05 are highlighted. See also Dataset S3.

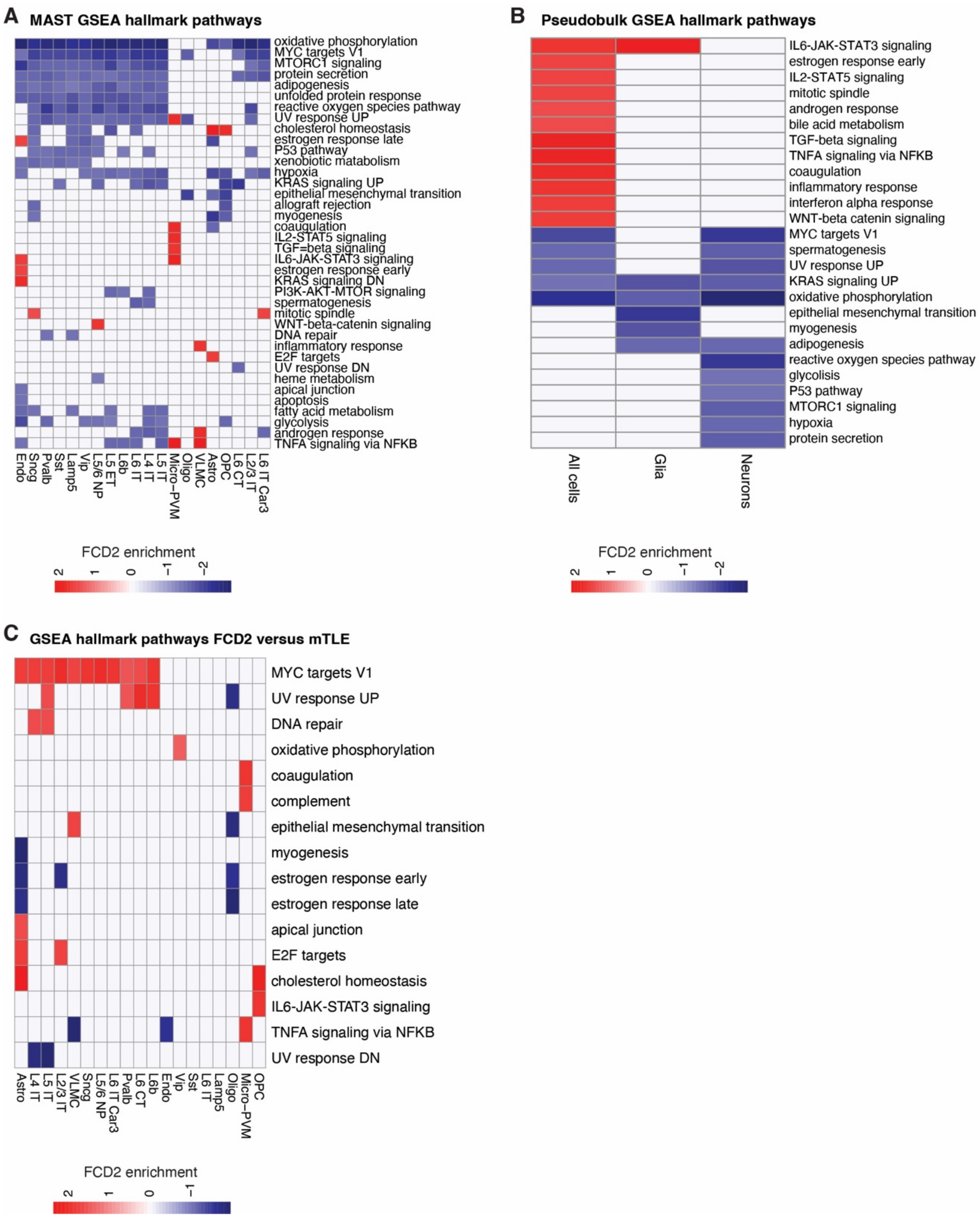

Fig. S10. (**A**) Heatmap showing Molecular Signatures Database Hallmark pathways (22, 23) positively (red) and negatively (blue) enriched in cases compared to controls per cell type, obtained through GSEA performed with fgsea (19) on differential expression analysis results obtained from MAST (24) (see also Dataset S4). (**B**) Heatmap showing Molecular Signatures Database Hallmark pathways positively (red) and negatively (blue) enriched in cases compared to controls per pseudobulk group, obtained through GSEA performed with fgsea on differential

expression analysis results obtained from Wilcox (see also Dataset S5). (**C**) Heatmap showing Molecular Signatures Database Hallmark pathways positively (red) and negatively (blue) enriched in cases compared to mTLE controls per cell type, obtained through GSEA performed with fgsea (see also Dataset S6 and Dataset S7).

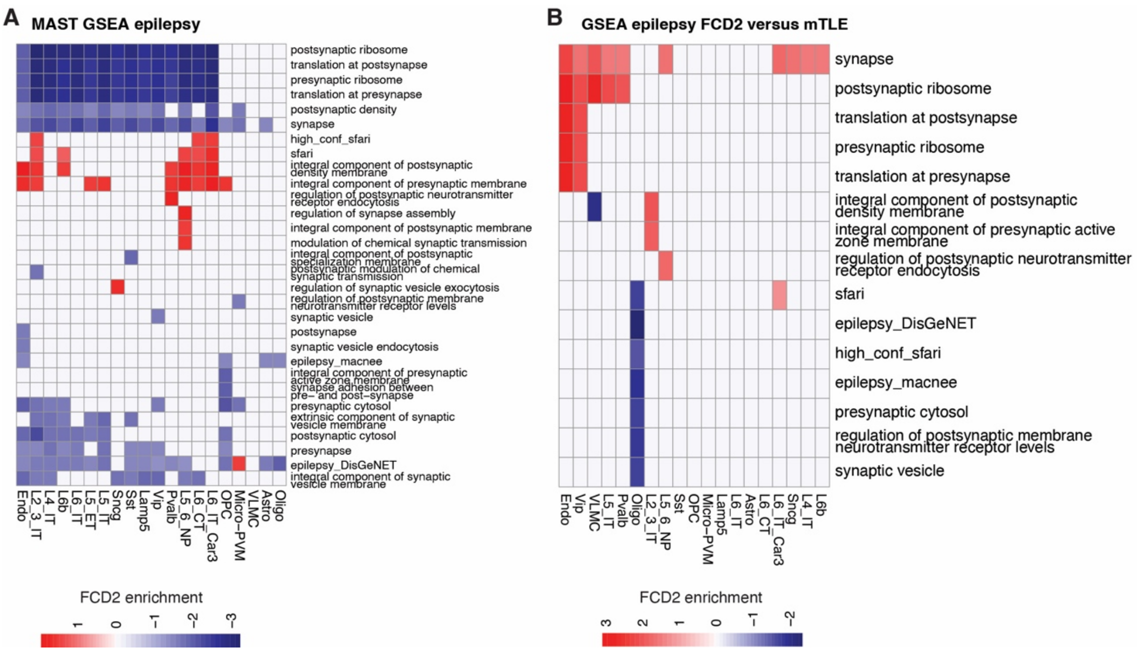

Fig. S11. (**A**) Heatmap showing pathways positively (red) and negatively (blue) enriched in cases compared to controls per cell type, obtained through GSEA performed with fgsea and epilepsy-related ontologies (SynGO (25), Macnee et al. (26), DisGeNET (27) and SFARI (28)) on differential expression analysis results obtained from MAST. (**B**) Heatmap showing pathways positively (red) and negatively (blue) enriched in cases compared to mTLE controls per cell type, obtained through GSEA performed with fgsea and epilepsy-related ontologies.

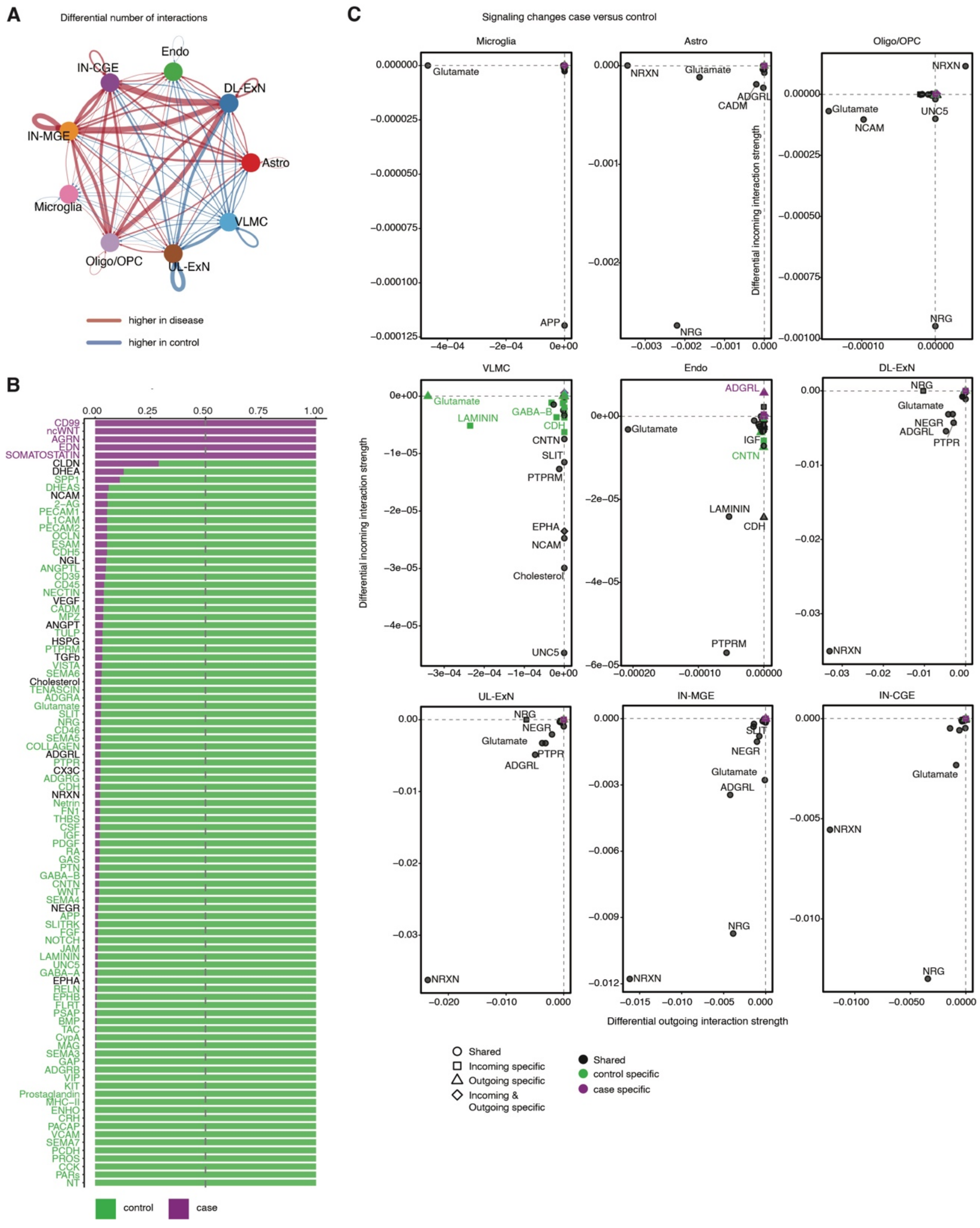

Fig. S12. (**A**) Circos plot where edge thickness represents the case versus control differential number of interactions predicted by CellChat (11). (**B**) Bar plot displaying the case versus control relative information flow predicted by CellChat for various curated human signaling pathways. (**C**) Dot plots displaying signaling changes identified by CellChat in case versus control nuclei.

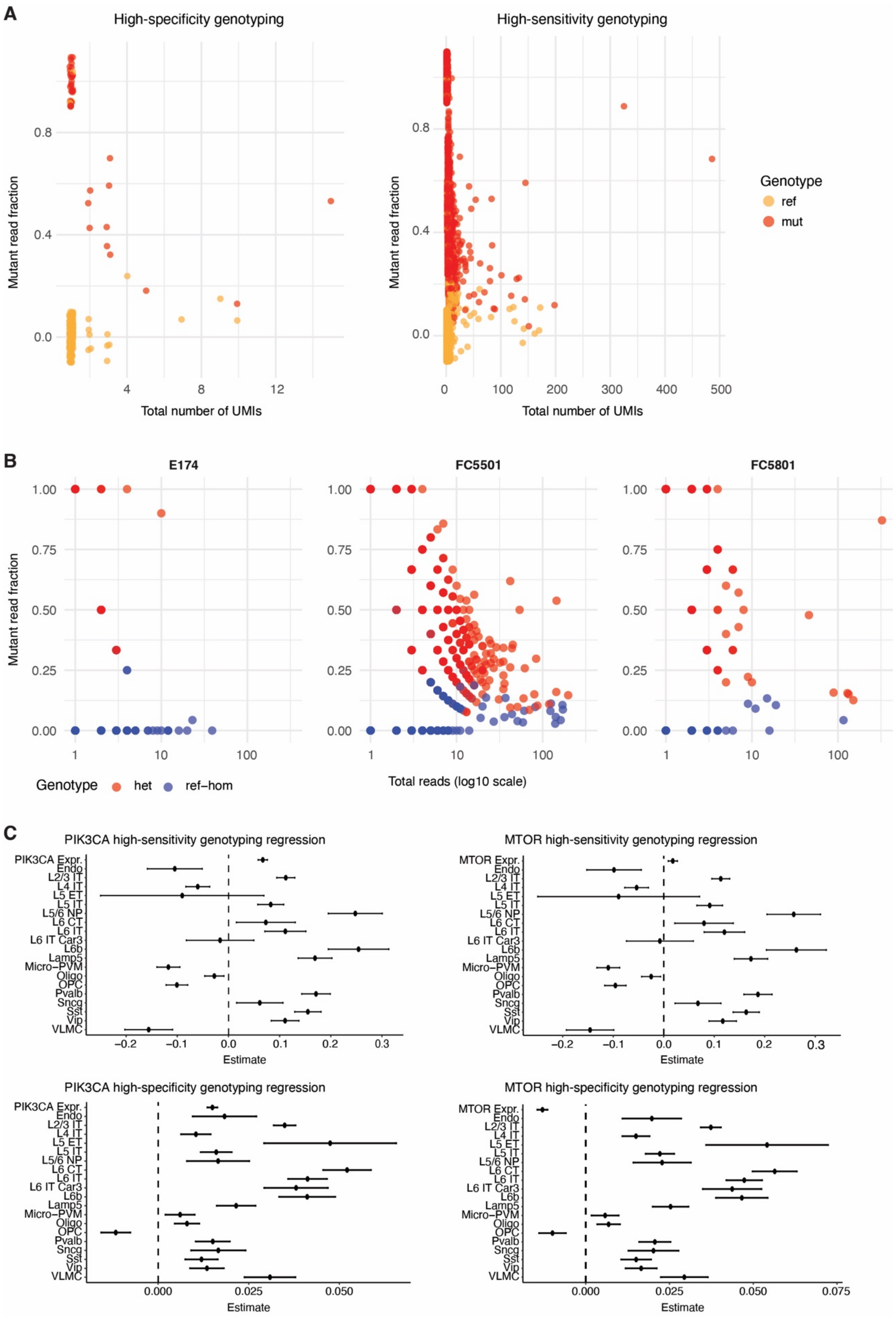
A
High-specificity genotyping
High-sensitivity genotyping
Mutant read fraction
Total number of UMIs
Genotype
ref
mut
B
E174
FC5501
FC5801
Total reads (log10 scale)
Genotype
het
ref–hom
C
PIK3CA high-sensitivity genotyping regression
MTOR high-sensitivity genotyping regression
PIK3CA high-specificity genotyping regression
MTOR high-specificity genotyping regression
PIK3CA Expr.
MTOR Expr.
Endo
L2/3 IT
L4 IT
L5 ET
L5 IT
L5/6 NP
L6 CT
L6 IT
L6 IT Car3
L6b
Lamp5
Micro–PVM
Oligo
OPC
Pvalb
Sncg
Sst
Vip
VLMC
Estimate

Fig. S13. (**A**) Total number of UMIs and mutant read fraction (proportion of reads that are mutant) distributions per genotyped nucleus for high-specificity and high-sensitivity GO-TEN analysis. (**B**) Mutant read fraction (number of reads with alternate allele/total number of reads) per nucleus, colored by final genotyping call. Data refers to the high-sensitivity genotyping approach. (**C**) Association of technical and biological covariates with GO-TEN high-sensitivity and high-specificity genotyping efficiency for *PIK3CA* and *MTOR* genes (logistic regression).

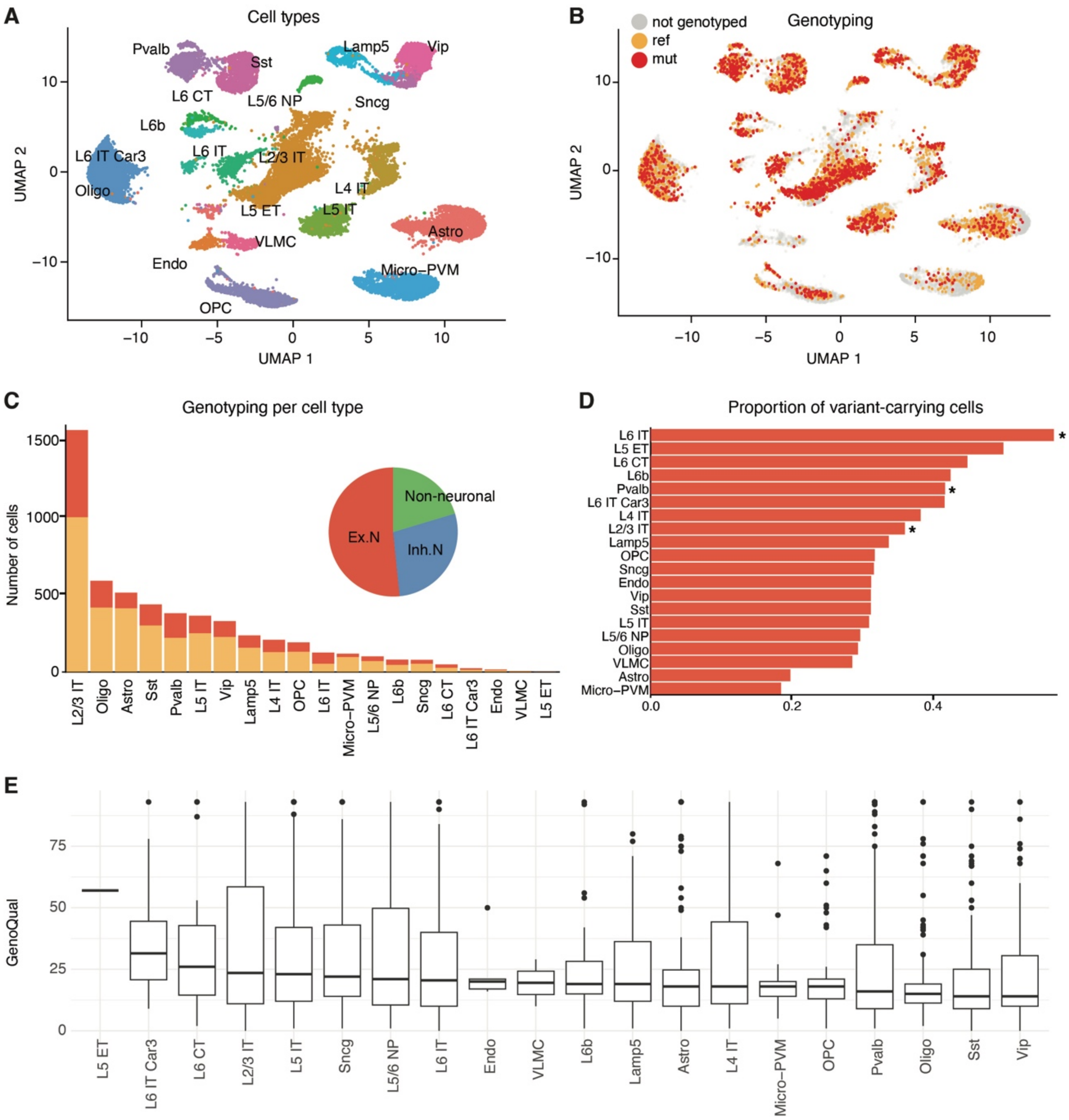

Fig. S14. (**A**) UMAP representation of cell types in integrated snRNA-seq atlas. (**B**) UMAP representation of *PIK3CA* and *MTOR* GO-TEN genotyped nuclei, colored as reference (ref) and mutant (mut). Data from three cases (one *PIK3CA* and two *MTOR*) were pooled together. Results refer to the high-sensitivity genotyping. (**C**) Number of nuclei genotyped with GO-TEN high-sensitivity approach as ref or mut. The pie chart shows the contribution of broad cell categories to variant-carrying nuclei. (**D**) Number of mut nuclei, normalized for the total number of genotyped nuclei for each variant-carrying cell type. Cell types enriched for mut nuclei are indicated with a * (hypergeometric test with Benjamini-Hochberg correction for multiple hypothesis testing, adj. $p < 0.05$). See also Dataset S14. (**E**) Genoqual scores of variant nuclei per cell type in which at least one variant nucleus was identified using the high-sensitivity genotyping approach. Cell types are sorted in descending order of median genoqual score.

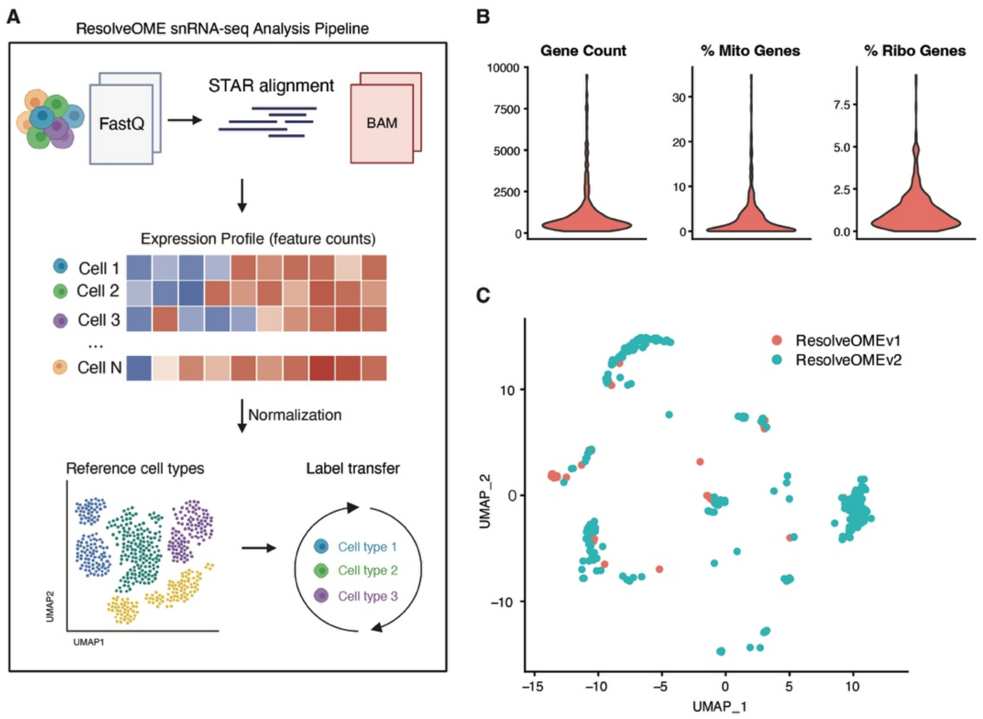

Fig. S15. (**A**) Schematic of the ResolveOME snRNA-seq pipeline. After mapping the raw fastq reads with STAR (16), gene count matrices were generated and normalized. For cell-type annotation, each nucleus was assigned a predicted cell type by mapping to the FCD2-only snRNA-seq reference dataset. (**B**) Overview of basic QC metrics including Gene Count, percent mitochondrial genes (% Mito Genes), and percent ribosomal genes (% Ribo Genes) in ResolveOME snRNA-seq data. (**C**) UMAP showing comparable distribution of snRNA-seq data generated using ResolveOME v1 (n=1 samples) and ResolveOME v2 (n=4 samples).

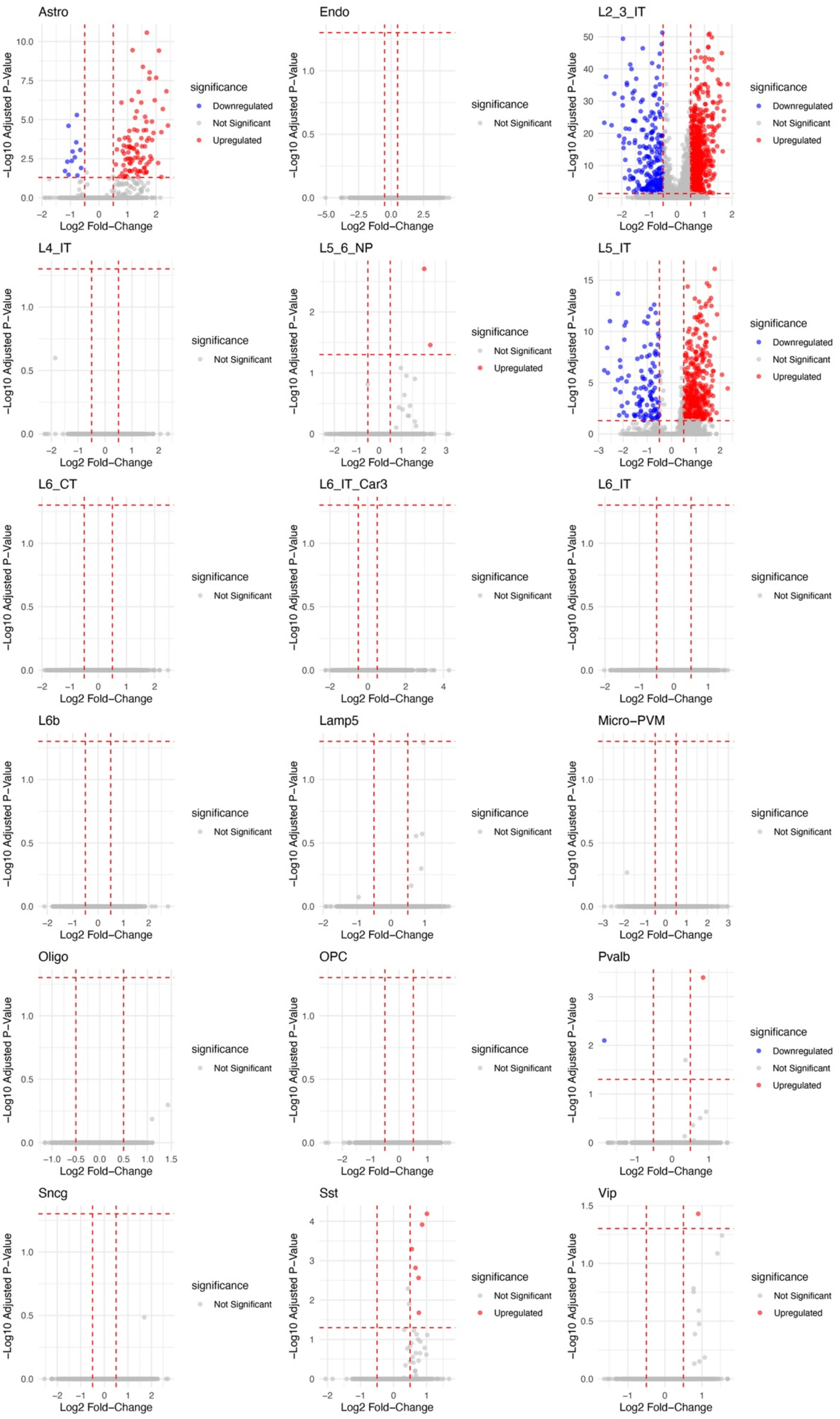
Astro
Endo
L2_3_IT
L4_IT
L5_6_NP
L5_IT
L6_CT
L6_IT_Car3
L6_IT
L6b
Lamp5
Micro−PVM
Oligo
OPC
Pvalb
Sncg
Sst
Vip
−Log10 Adjusted P−Value
Log2 Fold−Change
significance
Downregulated
Not Significant
Upregulated

Fig. S16. Volcano plots displaying cell-type-specific differentially expressed genes (DEGs) between mut and ref nuclei genotyped with GO-TEN high-sensitivity approach. Differential expression analysis was done with Seurat *FindMarkers* using a Wilcoxon rank sum test with a Benajmini-Hochberg correction for multiple hypothesis testing. DEGs with an average log-2 fold-change with absolute value > 0.5 and adjusted p-values < 0.05 are highlighted. See also Dataset S16.

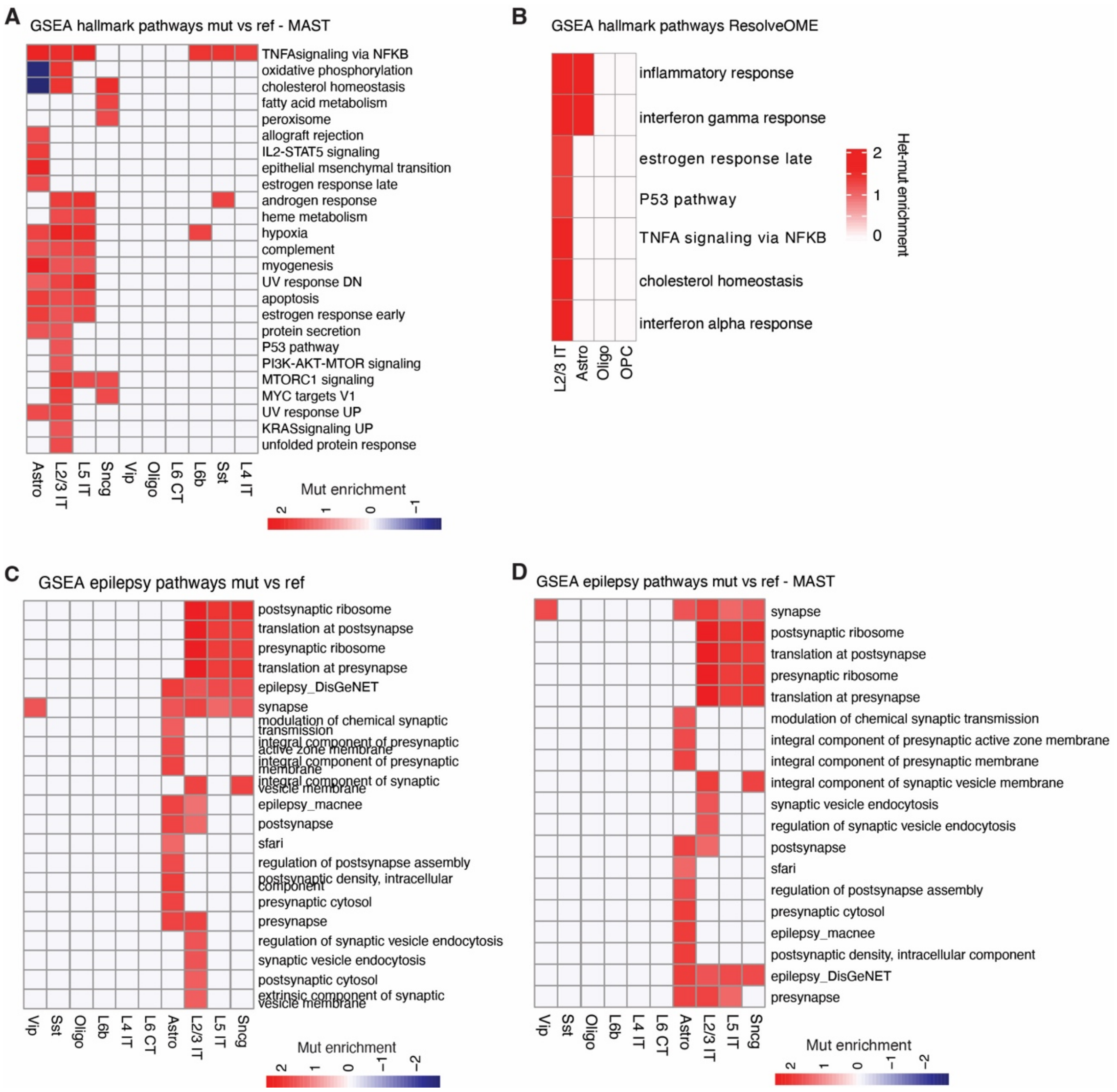

Fig. S17. (**A**) Heatmap showing Molecular Signatures Database Hallmark pathways positively (red) and negatively (blue) enriched in mut compared to ref GO-TEN genotyped nuclei per cell type, obtained through GSEA performed with fgsea on differential expression analysis results obtained from MAST (see also Dataset S17). High-sensitivity genotyping results were used for this analysis. (**B**) Heatmap showing Molecular Signatures Database Hallmark pathways positively (red) and negatively (blue) enriched in ResolveOME het-mut compared to ref-hom nuclei per cell type (adj. $p < 0.1$) obtained through GSEA performed with fgsea on differential expression analysis results obtained from Wilcoxon rank sum test and Benjamini-Hochberg correction (see also Dataset S18). (**C**) Heatmap showing Molecular Signatures Database Hallmark pathways positively (red) and negatively (blue) enriched in mut compared to ref controls per cell type, obtained through GSEA performed with fgsea. High-sensitivity calls were used for this analysis. (**D**) Heatmap showing cell-type-associated epilepsy-related pathways (SynGO, Macnee et al., DisGeNET and SFARI), positively (red) and negatively (blue) enriched in mut compared to ref GO-TEN genotyped nuclei per cell type, obtained through GSEA performed with fgsea on differential expression analysis results obtained from MAST. High-sensitivity calls were used for this analysis.

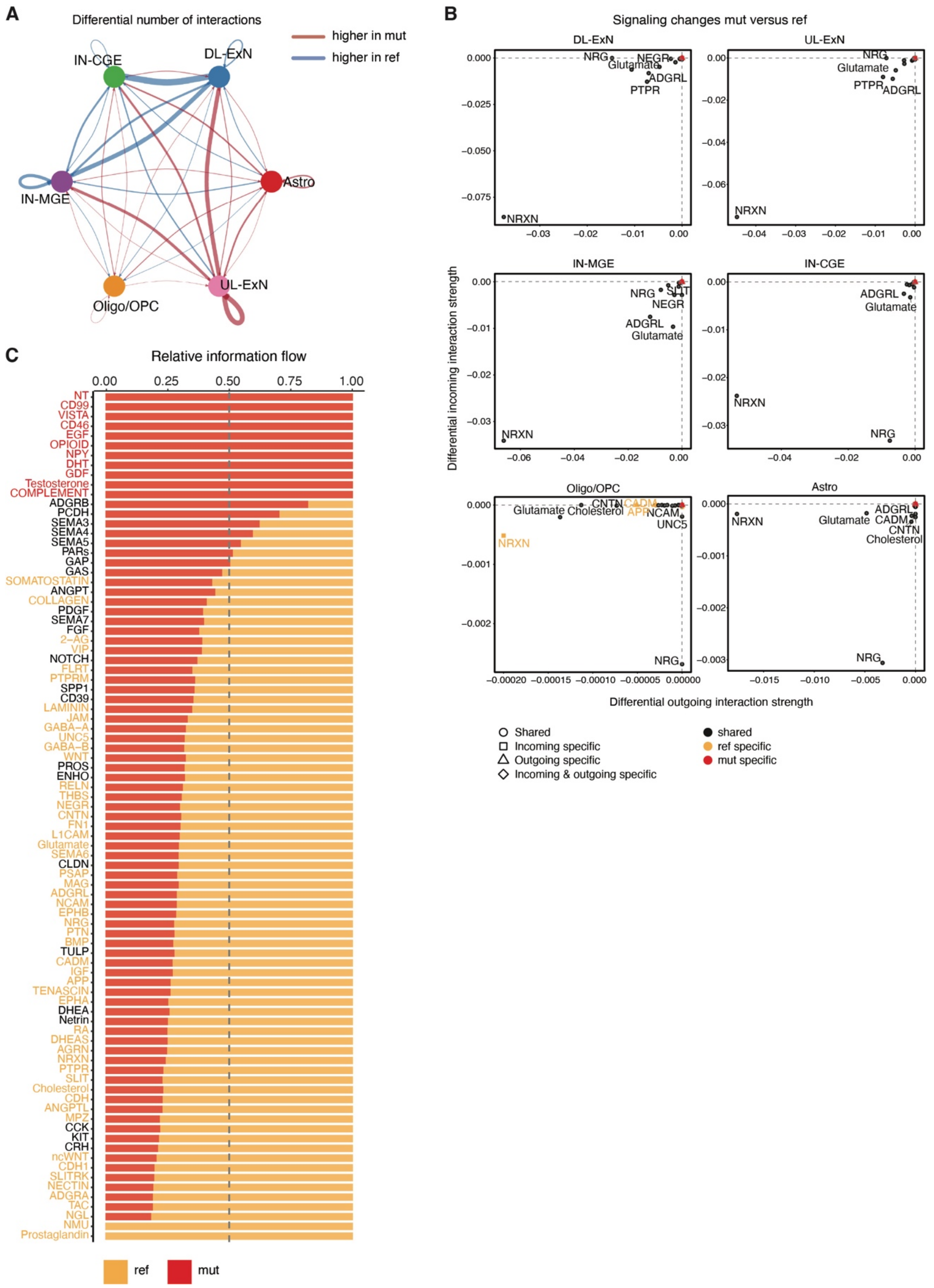
A
Differential number of interactions
higher in mut
higher in ref
IN-CGE
DL-ExN
IN-MGE
Astro
Oligo/OPC
UL-ExN
B
Signaling changes mut versus ref
Differential incoming interaction strength
Differential outgoing interaction strength
Shared
Incoming specific
Outgoing specific
Incoming & outgoing specific
shared
ref specific
mut specific
C
Relative information flow
ref
mut

Fig. S18. (**A**) Circos plot where edge thickness represents the GO-TEN mut versus ref differential number of interactions predicted by CellChat. High-sensitivity genotyping results were used for this analysis. (**B**) Bar plot displaying the mut versus ref relative information flow predicted by CellChat for each pathway. (**C**) Dot plots displaying signaling changes identified by CellChat in mut versus ref nuclei. See also Dataset S19.

Legends for Datasets S1 to S19

Dataset S1 (separate file). Additional clinical details related to the patient cohort included in the study.

Dataset S2 (separate file). Statistical analysis of the contribution of disease and control groups to different cell types done with propeller. Analysis refers to the plot in Fig. S3B.

Dataset S3 (separate file). Differentially expressed genes between FCD2 case and control groups for each cell type in the dataset calculated on snRNA-seq data. *P*-values and adjusted *p*-values correspond to the Wilcoxon rank sum test and Benjamini-Hochberg correction. Positive avg_log2FC values mean overexpression in FCD2. Corresponding GSEA analysis run with fgsea is also reported.

Dataset S4 (separate file). Differentially expressed genes between FCD2 case and control groups for each cell type in the dataset calculated on snRNA-seq data. *P*-values and adjusted *p*-values correspond to MAST and Benjamini-Hochberg correction. Positive avg_log2FC values mean overexpression in FCD2. Corresponding GSEA analysis run with fgsea is also reported.

Dataset S5 (separate file). Differentially expressed genes between FCD2 case and control groups for each pseudobulk group (all cells, glia, neurons) calculated on snRNA-seq data. *P*-values and adjusted *p*-values correspond to Wilcoxon rank sum test and Benjamini-Hochberg correction. Positive avg_log2FC values mean overexpression in FCD2. Corresponding GSEA analysis run with fgsea is also reported.

Dataset S6 (separate file). Differentially expressed genes between FCD2 case and mTLE control groups for each cell type in the dataset calculated on snRNA-seq data. *P*-values and adjusted *p*-values correspond to the Wilcoxon rank sum test and Benjamini-Hochberg correction. Positive avg_log2FC values mean overexpression in FCD2. Corresponding GSEA analysis run with fgsea is also reported.

Dataset S7 (separate file). Differentially expressed genes between FCD2 case and mTLE control groups for each cell type in the dataset calculated on snRNA-seq data. *P*-values and

adjusted *p*-values correspond to MAST and Benjamini-Hochberg correction. Positive avg_log2FC values mean overexpression in FCD2.

Dataset S8 (separate file). Results of CellChat analysis performed by comparing FCD2 cases with controls.

Dataset S9 (separate file). GO-TEN single-cell genotyping with cell type annotation.

Dataset S10 (separate file). GO-TEN read sequences for the high-sensitivity and high-specificity genotyping for case E174.

Dataset S11 (separate file). GO-TEN read sequences for the high-sensitivity and high-specificity genotyping for case FC5801.

Dataset S12 (separate file). GO-TEN read sequences for the high-specificity genotyping for case FC5501.

Dataset S13 (separate file). GO-TEN read sequences for the high-sensitivity genotyping for case FC5501.

Dataset S14 (separate file). *P*-values obtained for GO-TEN enrichment analysis using a hypergeometric test with Benjamini-Hochberg correction for multiple hypothesis testing. *P*-values refer to the plot in Fig. 4F.

Dataset S15 (separate file). ResolveOME single-cell ddPCR genotyping with cell type annotation.

Dataset S16 (separate file). Differentially expressed genes between mut and ref GO-TEN genotyped cells calculated on snRNA-seq data. *P*-values and adjusted *p*-values correspond to the Wilcoxon rank sum test and Benjamini-Hochberg correction. Positive avg_log2FC values mean overexpression in mut. Corresponding GSEA analysis run with fgsea is also reported.

Dataset S17 (separate file). Differentially expressed genes between mut and ref GO-TEN genotyped cells calculated on snRNA-seq data. *P*-values and adjusted *p*-values correspond to MAST and Benjamini-Hochberg correction. Positive avg_log2FC values mean overexpression in mut. Corresponding GSEA analysis run with fgsea is also reported.

Dataset S18 (separate file). Differentially expressed genes between het-mut and ref-hom ResolveOME genotyped cells. *P*-values and adjusted *p*-values correspond to the Wilcoxon rank sum test and Benjamini-Hochberg correction. Positive avg_log2FC values mean overexpression in het-mut.

Dataset S19 (separate file). Results of CellChat analysis performed by comparing mut with ref GO-TEN genotyped cells.

SI references

1. D. Lai, M. Gade, E. Yang, H. Y. Koh, J. Lu, N. M. Walley, A. F. Buckley, T. T. Sands, C. I. Akman, M. A. Mikati, G. M. McKhann, J. E. Goldman, P. Canoll, A. L. Alexander, K. L. Park, G. K. Von Allmen, O. Rodziyevska, M. B. Bhattacharjee, H. G. W. Lidov, H. Vogel, G. A. Grant, B. E. Porter, A. H. Poduri, P. B. Crino, E. L. Heinzen, Somatic variants in diverse genes leads to a spectrum of focal cortical malformations. *Brain* **145**, 2704-2720 (2022).
2. A. M. D'Gama, M. B. Woodworth, A. A. Hossain, S. Bizzotto, N. E. Hatem, C. M. LaCoursiere, I. Najm, Z. Ying, E. Yang, A. J. Barkovich, D. J. Kwiatkowski, H. V. Vinters, J. R. Madsen, G. W. Mathern, I. Blumcke, A. Poduri, C. A. Walsh, Somatic Mutations Activating the mTOR Pathway in Dorsal Telencephalic Progenitors Cause a Continuum of Cortical Dysplasias. *Cell Rep* **21**, 3754-3766 (2017).
3. C. Chung, X. Yang, T. Bae, K. I. Vong, S. Mittal, C. Donkels, H. Westley Phillips, Z. Li, A. P. L. Marsh, M. W. Breuss, L. L. Ball, C. A. B. Garcia, R. D. George, J. Gu, M. Xu, C. Barrows, K. N. James, V. Stanley, A. S. Nidhiry, S. Khoury, G. Howe, E. Riley, X. Xu, B. Copeland, Y. Wang, S. H. Kim, H. C. Kang, A. Schulze-Bonhage, C. A. Haas, H. Urbach, M. Prinz, D. D. Limbrick, Jr., C. A. Gurnett, M. D. Smyth, S. Sattar, M. Nespeca, D. D. Gonda, K. Imai, Y. Takahashi, H. H. Chen, J. W. Tsai, V. Conti, R. Guerrini, O. Devinsky, W. A. Silva, Jr., H. R. Machado, G. W. Mathern, A. Abyzov, S. Baldassari, S. Baulac, C. Focal Cortical Dysplasia Neurogenetics, N. Brain Somatic Mosaicism, J. G. Gleeson, Comprehensive multi-omic profiling of somatic mutations in malformations of cortical development. *Nat Genet* 10.1038/s41588-022-01276-9 (2023).
4. J. Ganz, L. J. Luquette, S. Bizzotto, M. B. Miller, Z. Zhou, C. L. Bohrson, H. Jin, A. V. Tran, V. V. Viswanadham, G. McDonough, K. Brown, Y. Chahine, B. Chhouk, A. Galor, P. J. Park, C. A. Walsh, Contrasting somatic mutation patterns in aging human neurons and oligodendrocytes. *Cell* **187**, 1955-1970 e1923 (2024).
5. K. Siletti, R. Hodge, A. Mossi Albiach, K. W. Lee, S. L. Ding, L. Hu, P. Lonnerberg, T. Bakken, T. Casper, M. Clark, N. Dee, J. Gloe, D. Hirschstein, N. V. Shapovalova, C. D. Keene, J. Nyhus, H. Tung, A. M. Yanny, E. Arenas, E. S. Lein, S. Linnarsson, Transcriptomic diversity of cell types across the adult human brain. *Science* **382**, eadd7046 (2023).
6. I. Korsunsky, N. Millard, J. Fan, K. Slowikowski, F. Zhang, K. Wei, Y. Baglaenko, M. Brenner, P. R. Loh, S. Raychaudhuri, Fast, sensitive and accurate integration of single-cell data with Harmony. *Nat Methods* **16**, 1289-1296 (2019).
7. Y. Hao, T. Stuart, M. H. Kowalski, S. Choudhary, P. Hoffman, A. Hartman, A. Srivastava, G. Molla, S. Madad, C. Fernandez-Granda, R. Satija, Dictionary learning for integrative, multimodal and scalable single-cell analysis. *Nat Biotechnol* **42**, 293-304 (2024).
8. T. E. Bakken, N. L. Jorstad, Q. Hu, B. B. Lake, W. Tian, B. E. Kalmbach, M. Crow, R. D. Hodge, F. M. Krienen, S. A. Sorensen, J. Eggermont, Z. Yao, B. D. Aevermann, A. I. Aldridge, A. Bartlett, D. Bertagnolli, T. Casper, R. G. Castanon, K. Crichton, T. L. Daigle, R. Dalley, N. Dee, N. Dembrow, D. Diep, S. L. Ding, W. Dong, R. Fang, S. Fischer, M. Goldman, J. Goldy, L. T. Graybuck, B. R. Herb, X. Hou, J. Kancherla, M. Kroll, K. Lathia, B. van Lew, Y. E. Li, C. S. Liu, H. Liu, J. D. Lucero, A. Mahurkar, D. McMillen, J. A. Miller, M. Moussa, J. R. Nery, P. R. Nicovich, S. Y. Niu, J. Orvis, J. K. Osteen, S. Owen, C. R. Palmer, T. Pham, N. Plongthongkum, O. Poirion, N. M. Reed, C. Rimorin, A. Rivkin, W. J. Romanow, A. E. Sedeno-Cortes, K. Siletti, S. Somasundaram, J. Sulc, M. Tieu, A. Torkelson, H. Tung, X. Wang, F. Xie, A. M. Yanny, R. Zhang, S. A. Ament, M. M. Behrens, H. C. Bravo, J. Chun, A. Dobin, J. Gillis, R. Hertzano, P. R. Hof, T. Hollt, G. D. Horwitz, C. D. Keene, P. V. Kharchenko, A. L. Ko, B. P. Lelieveldt, C. Luo, E. A. Mukamel, A. Pinto-Duarte, S. Preissl, A. Regev, B. Ren, R. H. Scheuermann, K. Smith,

W. J. Spain, O. R. White, C. Koch, M. Hawrylycz, B. Tasic, E. Z. Macosko, S. A. McCarroll, J. T. Ting, H. Zeng, K. Zhang, G. Feng, J. R. Ecker, S. Linnarsson, E. S. Lein, Comparative cellular analysis of motor cortex in human, marmoset and mouse. *Nature* **598**, 111-119 (2021).

9. M. D. Luecken, M. Büttner, K. Chaichoompu, A. Danese, M. Interlandi, M. F. Mueller, D. C. Strobl, L. Zappia, M. Dugas, M. Colomé-Tatché, F. J. Theis, Benchmarking atlas-level data integration in single-cell genomics. *Nature Methods* **19**, 41-50 (2022).

10. R. D. Hodge, T. E. Bakken, J. A. Miller, K. A. Smith, E. R. Barkan, L. T. Graybuck, J. L. Close, B. Long, N. Johansen, O. Penn, Z. Yao, J. Eggermont, T. Hollt, B. P. Levi, S. I. Shehata, B. Aevermann, A. Beller, D. Bertagnolli, K. Brouner, T. Casper, C. Cobbs, R. Dalley, N. Dee, S. L. Ding, R. G. Ellenbogen, O. Fong, E. Garren, J. Goldy, R. P. Gwinn, D. Hirschstein, C. D. Keene, M. Keshk, A. L. Ko, K. Lathia, A. Mahfouz, Z. Maltzer, M. McGraw, T. N. Nguyen, J. Nyhus, J. G. Ojemann, A. Oldre, S. Parry, S. Reynolds, C. Rimorin, N. V. Shapovalova, S. Somasundaram, A. Szafer, E. R. Thomsen, M. Tieu, G. Quon, R. H. Scheuermann, R. Yuste, S. M. Sunkin, B. Lelieveldt, D. Feng, L. Ng, A. Bernard, M. Hawrylycz, J. W. Phillips, B. Tasic, H. Zeng, A. R. Jones, C. Koch, E. S. Lein, Conserved cell types with divergent features in human versus mouse cortex. *Nature* **573**, 61-68 (2019).

11. S. Jin, C. F. Guerrero-Juarez, L. Zhang, I. Chang, R. Ramos, C. H. Kuan, P. Myung, M. V. Plikus, Q. Nie, Inference and analysis of cell-cell communication using CellChat. *Nat Commun* **12**, 1088 (2021).

12. H. Li, Minimap2: pairwise alignment for nucleotide sequences. *Bioinformatics* **34**, 3094-3100 (2018).

13. A. Y. Huang, Z. Zhang, A. Y. Ye, Y. Dou, L. Yan, X. Yang, Y. Zhang, L. Wei, MosaicHunter: accurate detection of postzygotic single-nucleotide mosaicism through next-generation sequencing of unpaired, trio, and paired samples. *Nucleic Acids Res* **45**, e76 (2017).

14. A. Y. Huang, P. Li, R. E. Rodin, S. N. Kim, Y. Dou, C. J. Kenny, S. K. Akula, R. D. Hodge, T. E. Bakken, J. A. Miller, E. S. Lein, P. J. Park, E. A. Lee, C. A. Walsh, Parallel RNA and DNA analysis after deep sequencing (PRDD-seq) reveals cell type-specific lineage patterns in human brain. *Proc Natl Acad Sci U S A* 10.1073/pnas.2006163117 (2020).

15. J. Lonsdale, J. Thomas, M. Salvatore, R. Phillips, E. Lo, S. Shad, R. Hasz, G. Walters, F. Garcia, N. Young, B. Foster, M. Moser, E. Karasik, B. Gillard, K. Ramsey, S. Sullivan, J. Bridge, H. Magazine, J. Syron, J. Fleming, L. Siminoff, H. Traino, M. Mosavel, L. Barker, S. Jewell, D. Rohrer, D. Maxim, D. Filkins, P. Harbach, E. Cortadillo, B. Berghuis, L. Turner, E. Hudson, K. Feenstra, L. Sobin, J. Robb, P. Branton, G. Korzeniewski, C. Shive, D. Tabor, L. Qi, K. Groch, S. Nampally, S. Buia, A. Zimmerman, A. Smith, R. Burges, K. Robinson, K. Valentino, D. Bradbury, M. Cosentino, N. Diaz-Mayoral, M. Kennedy, T. Engel, P. Williams, K. Erickson, K. Ardlie, W. Winckler, G. Getz, D. DeLuca, D. MacArthur, M. Kellis, A. Thomson, T. Young, E. Gelfand, M. Donovan, Y. Meng, G. Grant, D. Mash, Y. Marcus, M. Basile, J. Liu, J. Zhu, Z. Tu, N. J. Cox, D. L. Nicolae, E. R. Gamazon, H. K. Im, A. Konkashbaev, J. Pritchard, M. Stevens, T. Flutre, X. Wen, E. T. Dermitzakis, T. Lappalainen, R. Guigo, J. Monlong, M. Sammeth, D. Koller, A. Battle, S. Mostafavi, M. McCarthy, M. Rivas, J. Maller, I. Rusyn, A. Nobel, F. Wright, A. Shabalin, M. Feolo, N. Sharopova, A. Sturcke, J. Paschal, J. M. Anderson, E. L. Wilder, L. K. Derr, E. D. Green, J. P. Struewing, G. Temple, S. Volpi, J. T. Boyer, E. J. Thomson, M. S. Guyer, C. Ng, A. Abdallah, D. Colantuoni, T. R. Insel, S. E. Koester, A. R. Little, P. K. Bender, T. Lehner, Y. Yao, C. C. Compton, J. B. Vaught, S. Sawyer, N. C. Lockhart, J. Demchok, H. F. Moore, The Genotype-Tissue Expression (GTEx) project. *Nature Genetics* **45**, 580-585 (2013).

16. A. Dobin, C. A. Davis, F. Schlesinger, J. Drenkow, C. Zaleski, S. Jha, P. Batut, M. Chaisson, T. R. Gingeras, STAR: ultrafast universal RNA-seq aligner. *Bioinformatics* **29**, 15-21 (2013).

17. Y. Liao, G. K. Smyth, W. Shi, featureCounts: an efficient general purpose program for assigning sequence reads to genomic features. *Bioinformatics* **30**, 923-930 (2014).

18. M. Shi, Y. Yang, N. Huang, D. Zeng, Z. Mo, J. Wang, X. Zhang, R. Liu, C. Wang, X. Rong, Z. Wu, Q. Huang, H. Shang, J. Tang, Z. Wang, J. Cai, G. Huang, Y. Guan, J. Guo, Q. Mu, J. Wang, W. Liao, Genetic and microenvironmental evolution of colorectal liver metastases under chemotherapy. *Cell Rep Med* **5**, 101838 (2024).

19. G. Korotkevich, V. Sukhov, N. Budin, B. Shpak, M. N. Artyomov, A. Sergushichev, Fast gene set enrichment analysis. *bioRxiv* 10.1101/060012, 060012 (2021).

20. B. Phipson, C. B. Sim, E. R. Porrello, A. W. Hewitt, J. Powell, A. Oshlack, propeller: testing for differences in cell type proportions in single cell data. *Bioinformatics* **38**, 4720-4726 (2022).

21. S. L. Wolock, R. Lopez, A. M. Klein, Scrublet: Computational Identification of Cell Doublets in Single-Cell Transcriptomic Data. *Cell Syst* **8**, 281-291 e289 (2019).

22. A. Subramanian, P. Tamayo, V. K. Mootha, S. Mukherjee, B. L. Ebert, M. A. Gillette, A. Paulovich, S. L. Pomeroy, T. R. Golub, E. S. Lander, J. P. Mesirov, Gene set enrichment analysis: a knowledge-based approach for interpreting genome-wide expression profiles. *Proc Natl Acad Sci U S A* **102**, 15545-15550 (2005).

23. A. Liberzon, C. Birger, H. Thorvaldsdottir, M. Ghandi, J. P. Mesirov, P. Tamayo, The Molecular Signatures Database (MSigDB) hallmark gene set collection. *Cell Syst* **1**, 417-425 (2015).

24. G. Finak, A. McDavid, M. Yajima, J. Deng, V. Gersuk, A. K. Shalek, C. K. Slichter, H. W. Miller, M. J. McElrath, M. Prlic, P. S. Linsley, R. Gottardo, MAST: a flexible statistical framework for assessing transcriptional changes and characterizing heterogeneity in single-cell RNA sequencing data. *Genome Biology* **16**, 278 (2015).

25. F. Koopmans, P. van Nierop, M. Andres-Alonso, A. Byrnes, T. Cijsouw, M. P. Coba, L. N. Cornelisse, R. J. Farrell, H. L. Goldschmidt, D. P. Howrigan, N. K. Hussain, C. Imig, A. P. H. de Jong, H. Jung, M. Kohansalnodehi, B. Kramarz, N. Lipstein, R. C. Lovering, H. MacGillavry, V. Mariano, H. Mi, M. Ninov, D. Osumi-Sutherland, R. Pielot, K. H. Smalla, H. Tang, K. Tashman, R. F. G. Toonen, C. Verpelli, R. Reig-Viader, K. Watanabe, J. van Weering, T. Achsel, G. Ashrafi, N. Asi, T. C. Brown, P. De Camilli, M. Feuermann, R. E. Foulger, P. Gaudet, A. Joglekar, A. Kanellopoulos, R. Malenka, R. A. Nicoll, C. Pulido, J. de Juan-Sanz, M. Sheng, T. C. Sudhof, H. U. Tilgner, C. Bagni, A. Bayes, T. Biederer, N. Brose, J. J. E. Chua, D. C. Dieterich, E. D. Gundelfinger, C. Hoogenraad, R. L. Huganir, R. Jahn, P. S. Kaeser, E. Kim, M. R. Kreutz, P. S. McPherson, B. M. Neale, V. O'Connor, D. Posthuma, T. A. Ryan, C. Sala, G. Feng, S. E. Hyman, P. D. Thomas, A. B. Smit, M. Verhage, SynGO: An Evidence-Based, Expert-Curated Knowledge Base for the Synapse. *Neuron* **103**, 217-234 e214 (2019).

26. M. Macnee, E. Perez-Palma, J. A. Lopez-Rivera, A. Ivaniuk, P. May, R. S. Moller, D. Lal, Data-driven historical characterization of epilepsy-associated genes. *Eur J Paediatr Neurol* **42**, 82-87 (2023).

27. J. Pinero, J. M. Ramirez-Anguita, J. Sauch-Pitarch, F. Ronzano, E. Centeno, F. Sanz, L. I. Furlong, The DisGeNET knowledge platform for disease genomics: 2019 update. *Nucleic Acids Res* **48**, D845-D855 (2020).

28. B. S. Abrahams, D. E. Arking, D. B. Campbell, H. C. Mefford, E. M. Morrow, L. A. Weiss, I. Menashe, T. Wadkins, S. Banerjee-Basu, A. Packer, SFARI Gene 2.0: a community-

driven knowledgebase for the autism spectrum disorders (ASDs). *Mol Autism* **4**, 36 (2013).
